# oxPAPC‐Mediated lncRNA CYP1B1‐AS1 From Dendritic Cells Accelerates Atherosclerosis

**DOI:** 10.1111/jcmm.71066

**Published:** 2026-02-27

**Authors:** Yuheng Cheng, Lang Ni, Changhao Ke, Yuanjie He, Youyang Huang, Shiwan Lu, Yongchao Zhao, Junbo Ge, Bei Shi, Zhenglong Wang

**Affiliations:** ^1^ Zunyi Medical University Zunyi China; ^2^ Army Medical Center of People's Liberation Army (Daping Hospital) Chongqing China; ^3^ Department of Cardiology The Affiliated Hospital of Zunyi Medical University Zunyi China; ^4^ Department of Cardiology Zhongshan Hospital of Fudan University Shanghai China

**Keywords:** atherosclerosis, CYP1B1‐AS, dendritic cell, LncRNA, oxPAPC

## Abstract

Oxidised 1‐palmitoyl‐2‐arachidonoyl‐sn‐glycero‐3‐phosphorylcholine (oxPAPC), dendritic cells (DCs), and long non‐coding RNAs (lncRNAs) play crucial roles in atherosclerosis (AS). This study aimed to determine whether oxPAPC‐induced DC‐derived lncRNAs contribute to AS and to elucidate the underlying regulatory mechanisms. DCs were treated with increasing oxPAPC concentrations to assess transcriptomic changes. RNA sequencing was used to identify differential expression of lncRNAs. ChIP‐Seq and RNA pull‐down assays were used to assess direct binding between lncRNA CYP1B1‐AS1 and NFATC2. The association between *CYP1B1‐AS1* and *CYP1B1* was assessed using Pearson's correlation analysis. Elevated serum oxPAPC levels were confirmed in patients with coronary heart disease. In vitro, sustained oxPAPC stimulation activated the TLR4‐MD2 pathway in DCs. *CYP1B1‐AS1* was identified as the key oxPAPC‐induced DC‐derived lncRNA, with *Gm33055* as its murine homologue. RNA sequencing revealed oxPAPC‐driven alterations in DC chemotaxis, differentiation, and lymphocyte activation. Analysis of human atherosclerotic plaque‐derived DCs showed significant *CYP1B1‐AS1* upregulation. *Gm33055* enhanced *Cyp1b1* expression in murine DCs. Mechanistically, oxPAPC promoted NFATC2 nuclear translocation. NFATC2 binds to the *CYP1B1‐AS1* promoter, whereas *CYP1B1‐AS1* directly interacts with NFATC2, forming a positive regulatory loop. Adoptive transfer of m‐*CYP1B1‐AS1*‐expressing DCs into *Apoe* ^−/−^ mice accelerated AS progression. These findings identify a DC‐derived lncRNA‐mediated regulatory axis that promotes AS and suggest potential therapeutic targets.

## 1 Introduction

Atherosclerosis (AS), the leading cause of panvascular diseases, is broadly conceptualised by the “response‐to‐injury” hypothesis. Various factors induce vascular endothelial cell (VEC) injury and increase vascular permeability, facilitating lipid deposition, including oxidised low‐density lipoprotein (ox‐LDL), while concurrently promoting VEC expression of adhesion molecules that recruit monocytes/macrophages (M0‐Mφ) which further exacerbate VEC injury. Progressive lipid accumulation drives luminal narrowing, leading to arterial stenosis, ischemia, and hypoxia [1, 2, 3]. Although the specific triggers of VEC injury are diverse, downstream immune responses converge on common pathways. Therapeutic strategies targeting lipid modulation, blood pressure control, and inflammation have significantly improved clinical outcomes in patients with AS; nevertheless, significant challenges in AS prognosis persist. Further elucidation of AS pathogenesis is crucial to provide insights into disease mechanisms and advance diagnostic and therapeutic approaches.

Initially, innate immune cells recognise VEC injury through damage‐associated molecular patterns (DAMPs). These cells migrate, adhere, and infiltrate the intima to clear damaged tissue and deposited ox‐LDL [4, 5]. Persistent DAMP signalling promotes vascular inflammation. Immature dendritic cells (DCs) phagocytose damaged tissue and ox‐LDL and differentiate into mature DCs capable of activating naïve T cells. Mature DCs present apolipoprotein B100 (ApoB100) peptides to T cells via major histocompatibility complex class II (MHC‐II) molecules [6, 7], thereby shifting the immune response from innate to adaptive [6, 7]. Distinct T cell subsets secrete specific cytokines, defining the inflammatory microenvironment and ultimately determining atherosclerotic plaque fate (rupture, calcification, and fibrosis) [6, 8]. Although the T cell‐driven intimal inflammatory microenvironment is relatively uncontrollable, DCs—positioned upstream of T cells—serve as a critical link between innate and adaptive immunity, underscoring their pivotal role in AS progression.

Oxidised 1‐palmitoyl‐2‐arachidonyl‐sn‐glycero‐3‐phosphorylcholine (oxPAPC), generated at tissue injury sites [9, 10, 11, 12, 13], is a major bioactive component of ox‐LDL [14, 15] and an endogenous mimetic of the pathogen‐associated molecular pattern lipopolysaccharide (LPS). oxPAPC binds and promotes CD14 internalisation, competitively inhibiting toll‐like receptor 4 (TLR4) signalling [16, 17]. Conversely, oxPAPC binds the catalytic domain of caspase‐11 [16, 18] to maintain DC viability while inducing sustained interleukin‐1β (IL‐1β) release, a state termed DC hyperactivation [16, 17]. Although AS progression often involves ox‐LDL‐mediated TLR4 activation [19, 20, 21, 22], oxPAPC, a major ox‐LDL component, acts as a TLR4 antagonist [16, 17]. A proposed hypothesis suggests that in CD14‐deficient cells, oxPAPC enters the cytosol via endocytosis, binds to myeloid differentiation factor 2 (MD2), and subsequently activates the TLR4 pathway [17]. We hypothesised that within the chronic, recurrent immune inflammation of AS, oxPAPC depletes membrane CD14 on DCs, enters the cytosol, activates the TLR4 pathway, and induces a spectrum of alterations in DC biological activity.

Long non‐coding RNAs (lncRNAs), characterised by transcripts > 200 nucleotides lacking protein‐coding potential [23, 24], broadly regulate disease progression at epigenetic levels, including transcription, translation, and post‐translational modifications [24, 25, 26, 27]. Advances in high‐throughput sequencing and proteomics have enabled functional characterisation of lncRNAs, once considered genomic “dark matter.” Reported lncRNAs such as NEXN‐AS1, MANTIS, LeXis, MeXis, and MALAT1 modulate AS pathogenesis through mechanisms involving VEC repair, lipid regulation, and anti‐inflammatory effects [20, 28, 29, 30, 31]. Considering that oxPAPC induces DC hyperactivation and lncRNAs are closely implicated in AS progression, sustained oxPAPC stimulation likely alters the DC transcriptome and induces specific lncRNA expression. Furthermore, oxPAPC‐induced lncRNAs may be particularly relevant within the “response‐to‐injury” framework, exerting potent regulatory effects on AS progression.

In this study, we aimed to investigate how oxPAPC influences DC function via lncRNAs in the context of AS. We focused on identifying DC‐derived lncRNAs induced by oxPAPC and exploring their potential regulatory roles in DC chemotaxis, differentiation, and interaction with T cells. This work provides mechanistic insights into how oxPAPC modulates DC phenotypes and highlights potential targets for therapeutic intervention in AS.

## 2 Materials and Methods

### 2.1 Study Participants

Patients diagnosed with coronary heart disease were enrolled in this study according to established guidelines [32]. All human and animal protocols were approved by the local ethics committee, and written informed consent was obtained from all participants.

### 2.2 Cell Culture

In mice, 10 × 10^6^ bone marrow cells per well were cultured in 6‐well plates with 2 mL of complete RPMI 1640 medium (Hyclone, USA) supplemented with glutamine, penicillin–streptomycin (Solarbio, China), granulocyte–macrophage colony‐stimulating factor (GM‐CSF; 20 ng/mL, Peprotech, USA), and IL‐4 (10 ng/mL, Peprotech, USA). On day 2, the medium was replaced with fresh medium, and the cells were incubated at 37°C in a 5% CO_2_ atmosphere. On day 6, non‐adherent cells in the culture supernatant, along with loosely adherent cells harvested by gentle phosphate‐buffered saline (PBS) washing, were pooled and used as the starting material for most experiments. DCs were stimulated with LPS (1 μg/mL, Solarbio, China) for 2 h, followed by treatment with oxPAPC (50 μg/mL, InvivoGen, USA), ox‐LDL (100 μg/mL, Yiyuan Biotechnology, China), or a combination of oxPAPC (25 μg/mL) and ox‐LDL (50 μg/mL). Alternatively, DCs were continuously stimulated with LPS (1 μg/mL) for 48 h.

In humans, CD14^+^ monocytes were isolated via Ficoll density gradient centrifugation combined with CD14 magnetic beads (Metenyi, Germany). These cells were then cultured with GM‐CSF (20 ng/mL, Peprotech, USA) and IL‐4 (10 ng/mL, Peprotech, USA) for 6 days to induce monocyte‐derived DCs (moDCs).

### 2.3 Animal Experiments

Eight‐week‐old male *Apoe* ^*−/−*^ mice were purchased from Cavens (China) and randomly assigned to two groups: Control and ADV‐OE‐m‐CYP1B1‐AS1 ADV‐Con (*n* = 9 per group). ADV‐transfected DCs were intravenously injected into AS mice at a dose of 1 × 10^6^ cells/mouse. All the mice were continuously fed a high‐cholesterol diet (Xietong Pharmaceutical Bio‐engineering Co., China) for 12 weeks. Animal care and experimental procedures strictly adhered to the Guide for the Care and Use of Laboratory Animals (National Institutes of Health, USA) and complied with the Code of Ethics for Animal Experimentation of Zunyi Medical University.

### 2.4 Western Blot

Cells were lysed in RIPA buffer (Epizyme, China) containing 1% protease and phosphatase inhibitors, and protein samples were separated using sodium dodecyl sulfate‐polyacrylamide gel electrophoresis (SDS‐PAGE). Following gel electrophoresis, proteins were transferred onto polyvinylidene fluoride membranes (Epizyme, China). The membranes were blocked with 5% bovine serum albumin (Epizyme, China) in 1X PBS with Tween‐20 at 20°C–25°C for 2 h and then incubated overnight at 4°C with primary antibodies against GAPDH (HUABIO, ET1601‐4), CD14 (HUABIO, ET1610‐85), TLR4 (Proteintech, 19811‐AP), MyD88 (Abbkine, ABP0102), p65 (HUABIO, ET1603‐12), Cleaved‐Caspase‐1 (CST, 89332), ASC (ImmunoWay, YT0365), NLRP3 (HUABIO, ET1610‐93), ApoB (ImmunoWay, YT7819), and CYP1B1 (Proteintech, 18505‐1‐AP). Membranes were then incubated with a secondary antibody at room temperature for 1 h. Protein bands were visualised and detected using a chemiluminescent imaging system (Bio‐Rad, Chemidoc).

### 2.5 Enzyme‐Linked Immunosorbent Assay (ELISA)

ELISA kits were purchased from Jiangsu Meimian (China) and operated strictly according to the manufacturer's instructions.

### 2.6 FISH

FISH kits and probes were purchased from RiboBio (R11060.7; China). The experimental cell lines included human peripheral blood moDCs and mouse bone marrow‐derived DCs. After processing the cells according to the provided protocol, confocal microscopy was used to observe probe distribution.

### 2.7 Flow Cytometry

In the cell experiments, the media from different groups were thoroughly washed off with PBS, and DC concentrations were adjusted to 10^6^/100 μL. Antibodies (CD11c‐PE, CD11c‐FITC, CD14‐FITC, CD11b‐PE, CD11b‐FITC, A/I‐E‐FITC, CD80‐FITC, CD86‐FITC, TLR‐4/MD2‐FITC; all antibodies were purchased from BioLegend, USA) were added, and the cells were incubated at 4°C for 30 min in the dark. For peripheral blood collection, the mice were fully anaesthetised and heparinised, and blood was drawn from the apex of the heart. Peripheral blood mononuclear cells were isolated using a lymphocyte separation solution, and subsequent procedures followed the same protocol as for cell experiments.

### 2.8 RNA Isolation and Reverse Transcription Quantitative Polymerase Chain Reaction (RT‐qPCR)

Total RNA was isolated from cells or tissues using TRIzol reagent (Invitrogen, 15596026) according to the manufacturer's protocol. cDNA was synthesised using a reverse transcription kit (Absin, abs60246). RT‐qPCR was performed using the RealStar Green Fast Mixture containing ROX (Absin, abs601511). mRNA and lncRNA levels were normalised to those of GAPDH. The primer sequences used in this study are listed in Table 1.

**TABLE 1:** Primer sequences of RT‐qPCR.

RNA	Forward‐sequences	Reserve‐sequences
Gm33055	GCAGGGCTCACAGTTCCTCAATC	GCAGGTCTCAGTCATCAAGCAAGG
CYP1B1	GCCTCTTTCCGTGTGGTGTCTG	CTCCGCATCGTCGTGGTTGTAC
CD14	GTGTGCTTGGCTTGTTGCTGTTG	AGGGCTCCGAATAGAATCCGACTAG
Caspase‐11	ACACCAGACATCAGACAGCACATTC	CACATTTCTCCAGAGTTCCCACCTC
Caspase‐1	GCCGTGGAGAGAAACAAGGAGTG	CTATCAGCAGTGGGCATCTGTAGC
GAPDH	TCACCATCTTCCAGGAGCGAGAC	TGAGCCCTTCCACAATGCCAAAG

### 2.9 In Vitro Transcription–Translation Assay

The upstream Gm33055 plasmid containing the T7 promoter was purchased from Tsingke Bio (China), and first‐generation sequencing of the plasmid was performed by Tsingke Bio. The plasmid was linearised using EcoRI (Thermo Fisher, USA), and DNA purity was confirmed by agarose gel electrophoresis. In vitro transcription was performed using the TnT Quick Coupled Transcription/Translation System (L1170, Promega, USA). The transcription system was prepared according to the manufacturer's instructions, with enzyme‐free sterile water as the negative control and luciferase as the positive control. After terminating the reaction, the mixture was diluted fivefold, and band distribution was analysed using SDS‐PAGE followed by Coomassie Brilliant Blue staining.

### 2.10 RNA‐Seq

Fastp software (v0.20.0) was used to trim adapters and remove low‐quality reads to generate high‐quality clean reads, which were aligned to the human reference genome (hg38) using STAR software (v2.7.9a). For mRNA analysis, raw gene‐level mRNA read counts were obtained using featureCounts software (v2.0) to construct mRNA expression profiles. Differential expression analysis was performed using the edgeR software (v3.32.1) to calculate fold changes and *p*‐values, with mRNA annotations derived from the Ensembl GTF gene annotation database (v104). Gene Ontology (GO) and Kyoto Encyclopedia of Genes and Genomes (KEGG) pathway enrichment analyses were carried out using the clusterProfiler R package (v3.18.1) based on differentially expressed mRNAs. HTSeq software (v0.13.5) was used to obtain raw transcript‐level lncRNA read counts, which were then used to generate lncRNA expression profiles. Similar to the mRNA analysis, differential expression was assessed using edgeR (v3.32.1), with lncRNA annotations sourced from the Ensembl human GTF gene annotation database (v104). GO and KEGG pathway enrichment analyses for lncRNAs were performed with the clusterProfiler R package (v3.18.1), based on the nearby mRNAs of differentially expressed lncRNAs.

### 2.11 Cell Migration Assay

A Transwell assay was used to evaluate DC migration. A 24‐well Boyden chamber with a porous polycarbonate membrane (8 μm pore size; Corning, USA) was used to assess cell migration. Cells under different treatment conditions were seeded in the upper chamber (100 μL/well, 5 × 10^4^ cells/mL), while the lower chamber was filled with RPMI 1640 culture medium supplemented with 10% FBS (500 μL/well). After a 12‐h incubation (5% CO_2_, 37°C), cells that had migrated through the membrane were fixed with 4% paraformaldehyde for 15 min and stained with crystal violet (Solarbio, C8470) for 30 min. The migrated cells were photographed and counted under a microscope.

### 2.12 ChIP‐Seq

ChIP high‐throughput sequencing was performed using Newcore Biotechnology (Shanghai, China). ChIP was performed following ENCODE‐recommended protocols using an NFAT1 antibody (CST, 5861T) for ChIP‐Seq. The yield of the ChIP‐enriched DNA was quantified using the Quant IT fluorescence assay (Life Technologies, USA). Illumina sequencing libraries were prepared using the ND607 (Vazyme) DNA Library Prep Kit, according to the manufacturer's instructions. Library quality was assessed using the Agilent 2100 Bioanalyzer (Agilent, USA), followed by sequencing in paired‐end (PE) 150 mode on an Illumina NovoSeq 6000 sequencer, in accordance with the manufacturer's protocol. Bioinformatic analysis was conducted by Newcore Biotechnology (Shanghai, China). Fastp software (v0.20.0) was used to trim adapters and remove low‐quality reads to generate high‐quality clean reads. Clean reads were aligned to the reference genome using Bowtie2 software (v2.2.4). Peak calling was performed using MACS2 software (v2.2.7.1), and peak annotation was conducted using ChIPSeeker software (v1.30.3) based on GTF annotation files. Motifs were identified using the Homer software (v4.11), and differentially enriched regions were analysed using the MAnorm2 software (v1.2.0).

### 2.13 Tandem Mass Tag (TMT) Quantitative Proteome

Nuclear proteins from DC were isolated using a nucleus extraction kit (Abbkine, KTP3002) following different treatments (control and oxPAPC). TMT analysis was performed using the Wekemo software (Shenzhen, China).

### 2.14 RNA Pulldown

In vitro RNA transcription, RNA biotinylation, and protein capture from DC protein lysates (following oxPAPC stimulation) were performed according to the manufacturer's instructions using the T7 Quick High Yield RNA Transcription Kit (Beyotime, R7016S) and Pierce Magnetic RNA–Protein Pull‐Down Kit (Thermo Fisher, 20164). Positive and negative controls provided in the RNA pull‐down kit were included as corresponding controls.

### 2.15 Single‐Cell Transcriptome Analysis From the Gene Expression Omnibus (GEO) Database

The following AS‐related single‐cell sequencing datasets were obtained from the GEO database: GSE131778 [33], GSE155512 [34], GSE159677 [35], GSE196943 [36], GSE233870 [37], GSE234077 [38], and GSE260657 [39]. A total of 57 cases from the single‐cell RNA sequencing (scRNA‐Seq) datasets were integrated using Seurat (v5.1.0). The 10× scRNA‐Seq read matrices, representing human AS backgrounds, were merged using the merging function in R (v4.2.2). Cells with RNA counts greater than 40,000, feature RNAs between 500 and 5000, and mitochondrial RNA content > 10% were filtered out to exclude potential doublets and dead cells. Batch effects were mitigated by applying Seurat in combination with Harmony (v1.2.1), and cell clusters were identified for *PTPRC* ^+^ *CYP1B1* ^+^ cells using standard Seurat workflows. Differentially expressed genes were determined for each cell population, and differential signalling pathways between different cell types were analysed using CellChat.

### 2.16 Weighted Gene Co‐Expression Network Analysis (WGCNA) and Network Visualisation

WGCNA was performed to identify genes co‐expressed with CYP1B1. Samples were screened for outliers, and a soft‐threshold power was selected based on scale‐free topology (*R* ^2^ > 0.85). Modules were defined by hierarchical clustering of the topological overlap matrix (TOM). Network visualisation of module genes was performed using Cytoscape (v3.8.0). Module annotation and pathway enrichment analyses were carried out as described in the RNA‐Seq section.

### 2.17 Statistical Analysis

Statistical analysis was conducted using SPSS 29.0 statistical software (v29.0; IBM Corp., Armonk, NY, USA), GraphPad Prism 9, and R software. Statistical significance was set at *p* < 0.05. Normality of the measurement data was assessed using the Shapiro–Wilk test or Levene test. Non‐normally distributed data were presented as medians, and the non‐parametric rank‐sum test was employed to evaluate differences. Normally distributed data were expressed as means ± standard deviations, and the independent samples *t*‐test was utilised to compare means between the two groups.

## 3 Results

### 3.1 oxPAPC Stimulation Upregulated lncRNA CYP1B1‐AS1 in DC

**FIGURE 1:**
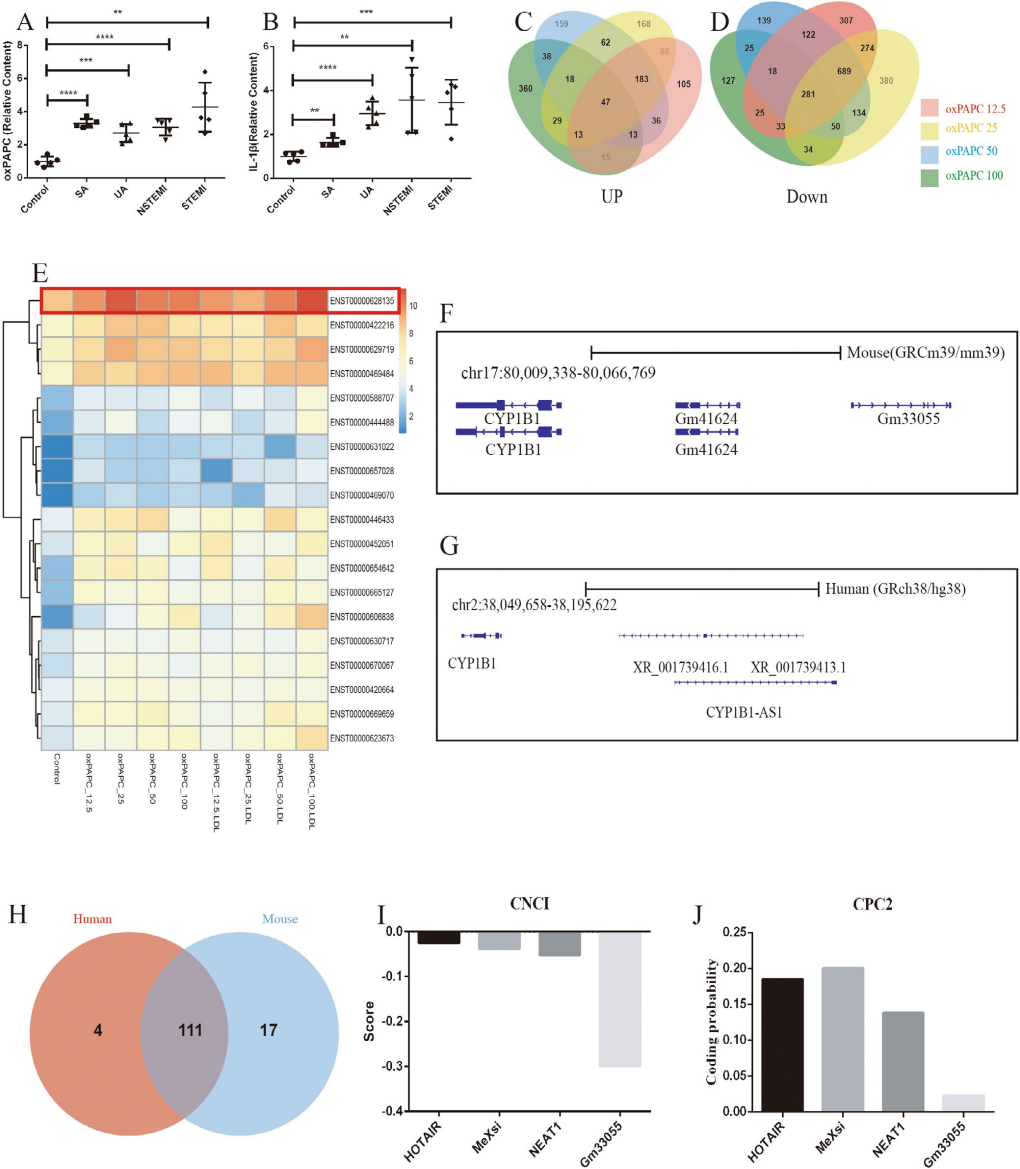
The screening and evaluation of DC‐derived lncRNA. (A, B) The plasma levels of oxPAPC and IL‐1β in CHD. (C) The up‐regulated lncRNAs under various oxPAPC stimuli. (D) The down‐regulated lncRNAs under various oxPAPC stimuli. (E) The expression level of the ENST00000628135 (CYP1B1‐AS1). (F) The genomic localisation of mouse for Gm33055 and CYP1B1‐AS1 by Integrative Genomics Viewer (IGV). (G) The genomic localisation of human for Gm33055 and CYP1B1‐AS1 by IGV. (H) The intersection of Gm33055 and RNA‐binding proteins in the promoter region of CYP1B1‐AS1 by RBP map analysis. (I) The non‐coding potential of Gm33055 by Coding‐Non‐Coding Index (CNCI) prediction. (J) The non‐coding potential of Gm33055 by Coding Potential Calculator 2 (CPC2) prediction (**P < 0.01, ***P < 0.001, ****P < 0.0001).

This study enrolled 30 patients undergoing angiography at the Department of Cardiovascular Medicine, Affiliated Hospital of Zunyi Medical University. Baseline clinical characteristics are summarised in Table 2. Plasma IL‐1β and oxPAPC levels were significantly elevated in patients with coronary artery disease compared with healthy controls (Figure 1A,B). DCs treated with increasing oxPAPC concentrations showed significant transcriptomic changes, identifying 47 upregulated and 281 downregulated lncRNAs (Figure 1C,D, Table 3). CYP1B1‐AS1 (ENST000000628135) was dose‐dependently upregulated following oxPAPC stimulation (Figure 1E).

**TABLE 2:** General clinical data of patients.

Items	Control	SA	UA	NSTEMI	STEMI
Age (years)	45.67 ± 0.58	53.24 ± 5.42	66.67 ± 5.69	57.33 ± 17.21	63.33 ± 19.55
Culprit vessel (branch)	0 ± 0	1 ± 0.68	2 ± 0	2 ± 1	2.33 ± 0.58
Systolic blood pressure (mmHg)	117 ± 6.25	114.62 ± 11	127.33 ± 23.59	146.67 ± 30.55	142.33 ± 15
Diastolic blood pressure (mmHg)	76.33 ± 3.79	68.45 ± 5.63	81.33 ± 5.69	73.33 ± 15.50	78.33 ± 7.64
Heart rate (per min)	77 ± 9.17	84 ± 6.24	84 ± 13.53	73.33 ± 15.50	77 ± 8.54
WBC (^10/L)	8.45 ± 4.40	6.52 ± 2.41	8.7 ± 1.58	9.78 ± 3.74	9.62 ± 3.47
TG (mmol/L)	5.94 ± 0.61	3.78 ± 0.67	3.75 ± 0.55	4.63 ± 0.47	4.44 ± 1.06
TC (mmol/L)	2.68 ± 1.16	2.75 ± 1.24	1.22 ± 0.32	3.42 ± 2.10	2.89 ± 0.78
LDL‐C (mmol/L)	1.60 ± 0.21	1.58 ± 0.48	2.35 ± 0.58	2.77 ± 0.23	2.83 ± 0.78
HDL‐C (mmol/L)	1.39 ± 0.31	0.96 ± 0.10	1.09 ± 0.29	1.03 ± 0.15	1.04 ± 0.10
CK (U/L)	55.67 ± 2.52	52.67 ± 3.25	51.67 ± 23.80	623.67 ± 478.73	112.33 ± 29
CK‐MB (U/L)	19.67 ± 14	21.58 ± 12	11.67 ± 1.53	44 ± 30.27	21 ± 1.0
Hs‐cTnI (ng/L)	0.12 ± 0.03	0.45 ± 0.12	1.21 ± 1.86	927.75 ± 782.42	2799.95 ± 4797.84
BNP (pg/mL)	51.11 ± 21	78.54 ± 35	61.91 ± 5.47	623.03 ± 656.14	2432.44 ± 2209.07

**TABLE 3:** Statistics of differentially expressed mRNA and lncRNA.

Comparisons	mRNA			lncRNA		
	Up	Down	Total	Up	Down	Total
oxPAPC (12.5) vs. Control	798	3487	4285	500	1875	2375
oxPAPC (25) vs. Control	932	3551	4483	608	1749	2357
oxPAPC (50) vs. Control	725	3013	3738	556	1458	2014
oxPAPC (100) vs. Control	435	810	1245	533	593	1126
oxPAPC (12.5) + oxLDL vs. Control	896	3510	4406	1793	6547	8340
oxPAPC (25) + oxLDL vs. Control	274	1547	1822	1710	3066	4776
oxPAPC (50) + oxLDL vs. Control	755	3366	4121	2186	6159	8345
oxPAPC (100) + oxLDL vs. Control	489	887	1376	1785	2334	4119

### 3.2 Gm33055 Is the Functional Murine Homologue of Human CYP1B1‐AS1

UCSC LiftOver mapped the human CYP1B1‐AS1 locus (hg38, Chr2: 38,074,873–38,181,828) to the mouse mm39 genome, identifying *Gm33055* as a syntenic gene adjacent to *Cyp1b1* (Figure 1F,G). Accordingly, we first employed the UCSC Comparative Genomics track, which revealed the high CYP1B1‐AS1 promoter conservation across multiple species (Figure S1A). RNA‐binding protein analysis indicated > 85% overlap in predicted RBPs (Figure 1H). Regarding the coding potential of *Gm33055*, CPC2 and CNCI analyses predicted its coding probability to be even lower than that of established lncRNAs such as *HOTAIR*, *MeXis*, and *NEAT1* (Figure 1I,J). Although CYP1B1‐AS1 has been definitively annotated as an lncRNA in multiple databases, including NCBI, direct experimental evidence confirming the non‐coding nature of Gm33055 (m‐CYP1B1‐AS1) remains lacking, despite bioinformatic predictions. To address this, we conducted an in vitro transcription‐translation assay. The plasmid structure for T7‐m‐CYP1B1‐AS1‐pUC57 is shown in Figure S1E. Sanger sequencing verified the plasmid sequence (Figure S1B). Using T7‐firefly luciferase cDNA as a positive control and nuclease‐free water as a negative control, we observed that T7‐m‐CYP1B1‐AS1 exhibited identical banding patterns to the negative control (Figure S1C,D), confirming its non‐coding property. Based on these findings, we designated *Gm33055* as mouse CYP1B1‐AS1 (m‐CYP1B1‐AS1) and the retained CYP1B1‐AS1 as the human transcript (h‐CYP1B1‐AS1).

### 3.3 CYP1B1‐AS1 Promotes CYP1B1 Expression in DCs

**FIGURE 2:**
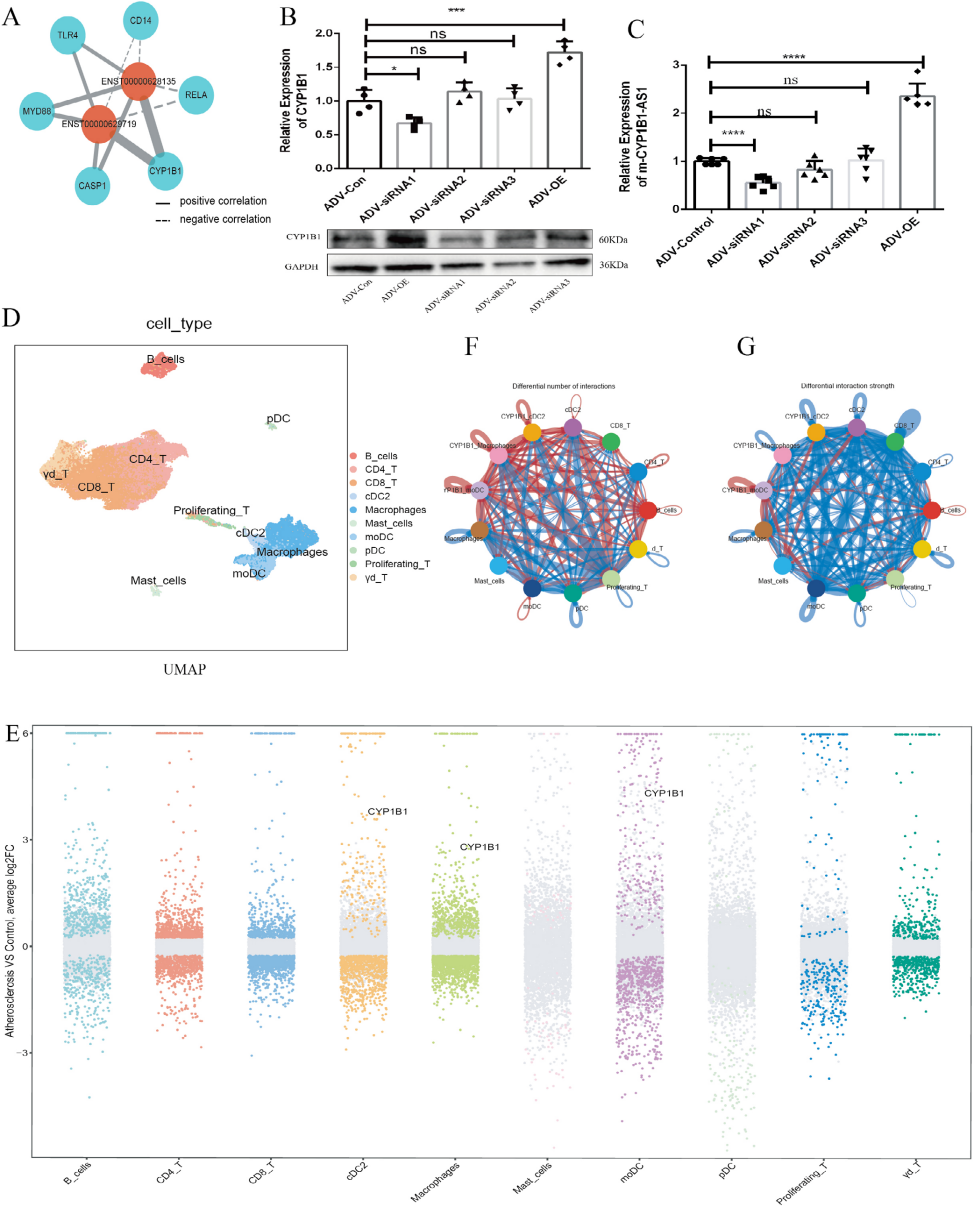
The relationship between CYP1B1‐AS1, CYP1B1 and AS. (A) Calculation of the Pearson correlation coefficient between CYP1B1 and CYP1B1‐AS1. (B) The CYP1B1 expression levels after knockdown and over‐expression of m‐CYP1B1‐AS1 by immunoblotting analysis. (C) The success of knockdown and over‐expression CYP1B1‐AS1 (m‐CYP1B1‐AS1) in mouse by PCR validation. (D) The subsets and distribution of PTPRC+ CYP1B1+ cells by Dimplot (Seurat). (E) The differentially expressed genes among cell subpopulations (Coloured point: Differentially expressed genes, log_2_FoldChange > 0.25 and adjusted *p*‐value < 0.01). (F, G) The differential number of interactions and interaction strength in CellChat (*p < 0.05, ***p < 0.001, ****p < 0.0001).

Pearson correlation analysis revealed a high correlation between CYP1B1‐AS1 and CYP1B1 expression (*r* = 0.97, Figure 2A), suggesting CYP1B1‐AS1 regulates DC phenotype by modulating CYP1B1. Adenovirus‐mediated knockdown and overexpression of m‐CYP1B1‐AS1 confirmed that CYP1B1‐AS1 promotes CYP1B1 expression (Figure 2B,C).

### 3.4 CYP1B1 Co‐Expression Network and Immune Relevance

Among the resulting modules, the cyan module exhibited the strongest correlation with *CYP1B1* (Figure S2B). Analysis of the cyan module revealed a strong association with transcriptional regulation and immune processes (Figure S2G), suggesting that under oxPAPC stimulation, *CYP1B1* acts upstream of key pathways shaping DC phenotype.

### 3.5 scRNA‐Seq Analysis of Atherosclerotic Lesions

Integration of 57 human AS lesions revealed that macrophages and DCs expressed high levels of CYP1B1 (Figures 2D,E and S3F). DCs showed higher CYP1B1 expression than other cell types (Figure S3E). CellChat analysis demonstrated that CYP1B1^+^ DCs engaged more interactions with proliferating T cells than CYP1B1^+^ macrophages, involving chemotaxis and inflammatory pathways (Figures 2F,G and S4–, S7).

### 3.6 Sustained oxPAPC Stimulation Activates the TLR4 Pathway Through CD14 Depletion, Affecting DC Differentiation, Chemotaxis, and Lymphocyte Proliferation

**FIGURE 3:**
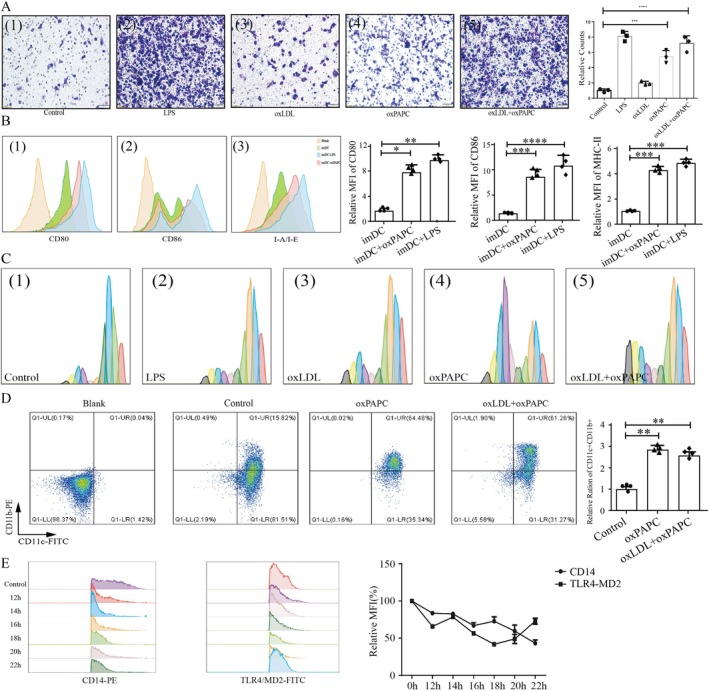
The effects of oxPAPC on DC, lymphocyte and CD14. (A) The chemotactic ability of DCs under oxPAPC stimulation by transwell assay. (B) The expression levels of CD80, CD86, and MHC‐II before and after oxPAPC/LPS stimulation by flow cytometry (FCM). (C) The changes in the ability of DC to stimulate lymphocyte proliferation by CFSE assay. (D) The analysis of the CD11b^+^ CD11c^+^ ratio by FCM. (E) The fluorescent signals of CD14 and TLR4‐MD2 on the surface of DC at different time under oxPAPC stimulation by flow cytometry (FCM) (*p < 0.05, **p < 0.01, ***p < 0.001, ****p < 0.0001).

**FIGURE 4:**
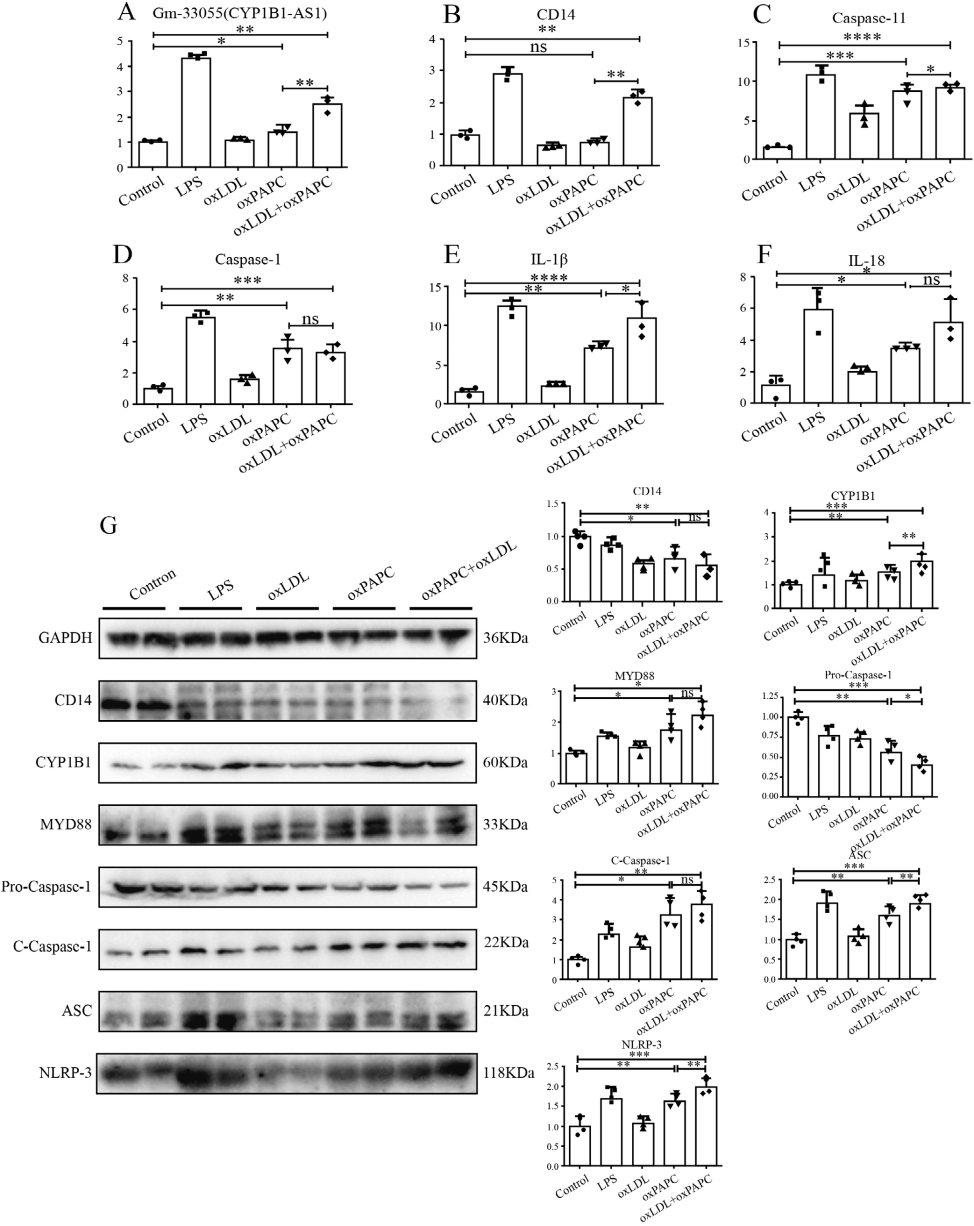
The expression levels of CD14, TLR‐4 signalling pathway and CYP1B1 by rt‐PCR and Western Blot. (A–F) The transcription levels of Gm33055, CD14, Caspase‐11, Caspase‐1, IL‐1β, and IL‐18 by rt‐PCR. (G) The expression levels of CD14, TLR‐4 signalling molecules, cell death‐related molecules, and CYP1B1 by Western Blot (*p < 0.05, **p < 0.01, ***p < 0.001, ****p < 0.0001).

We hypothesised that sustained oxPAPC stimulation would activate the TLR4 pathway following CD14 depletion. Defining CD14 and TLR4‐MD2 levels on unstimulated DC membranes as 100%, we monitored these signals every 2 h starting at 12 h post‐stimulation. TLR4‐MD2 signals decreased over the first 18 h, but subsequently increased concomitant with progressive CD14 depletion (Figure 3E). RT‐PCR analysis confirmed that oxPAPC did not induce the transcriptional upregulation of CD14 (Figure 4A–F), supporting the hypothesis of CD14 depletion. Western blotting further demonstrated CD14 depletion and an increased expression of TLR4 pathway‐associated molecules under sustained oxPAPC stimulation (Figure 4G). Given the crucial role of lymphocytes in AS progression [8, 40], we investigated the effect of oxPAPC‐sustained stimulation of DCs on lymphocyte proliferation. Initial analysis of the AS‐associated antigen ApoB100 in the NCBI datasets revealed its predominant hepatic expression, but not DC expression (Figure S8A). However, oxPAPC, ox‐LDL, and oxPAPC + ox‐LDL stimulation significantly increased ApoB levels in the DCs (Figure S8B). oxPAPC also markedly enhanced DC chemotaxis (Figure 3A) and upregulated DC surface expression of MHC‐II, CD80, and CD86 (Figure 3B). CFSE‐based lymphocyte proliferation assays confirmed that the oxPAPC‐treated DCs potently stimulated lymphocyte proliferation (Figure 3C). As CD11b^+^ CD11c^+^ DCs accelerated AS progression [41], oxPAPCs levels increased (Figure 3D). No significant changes in plasma oxPAPC levels were detected in the mouse left anterior descending artery ligation model of myocardial infarction (MI) (Figure S9B). Although IL‐1β increased by day 7 post‐MI, no elevation was observed in the early MI phase (Figure S9A). Similarly, early MI showed no changes in peripheral blood frequencies of CD11b^+^ CD11c^+^ cells or CD80/CD86 levels (Figure S9C,D). These phenotypic observations aligned with the significantly enriched GO terms and KEGG pathways identified in the differential mRNA analysis across oxPAPC concentrations (Tables 4 and 5).

**TABLE 4:** Common significant GO terms of upregulated differential mRNA under different oxPAPC.

GO ID	Annotation
GO:0001819	Positive regulation of cytokine production
GO:0046651	Lymphocyte proliferation
GO:0051090	Regulation of DNA‐binding transcription factor activity
GO:0001819	Positive regulation of cytokine production
GO:0032731	Positive regulation of interleukin‐1 beta production
GO:0022409	Positive regulation of cell–cell adhesion
GO:0050707	Regulation of cytokine secretion
GO:0006631	Fatty acid metabolic process
GO:0032755	Positive regulation of interleukin‐6 production

**TABLE 5:** Common pathways of differential genes under different oxPAPC stimulations.

Pathway ID	Annotation
hsa04514	Cell adhesion molecules (CAMs)
hsa04670	Leukocyte transendothelial migration
hsa04145	Phagosome
hsa04530	Tight junction
hsa04510	Focal adhesion
hsa04520	Adherens junction
hsa00030	Pentose phosphate pathway

### 3.7 DCs Overexpressing CYP1B1‐AS1 Accelerate AS

Histological assessment (haematoxylin and eosin, Masson's trichrome, Oil Red O staining) of ascending aortas from atherosclerotic mice receiving adoptively transferred DCs revealed that the ADV‐OE‐m‐CYP1B1‐AS1 group exhibited comparable fibrosis levels (Figure S10B,H,N,Q), but significantly larger atherosclerotic lesions (Figure S10A,D,G,J) and elevated intraplaque lipid content (Figure S10C,F,I,L) relative to controls. A similar pathological progression was observed in the aorta (Figure S10), demonstrating accelerated plaque development and lipid accumulation following DC transfer. Ultrastructural analysis showed that endothelial cells in the control group displayed an abnormal morphology with focal ribosomal loss, mild mitochondrial swelling, and slight rough endoplasmic reticulum dilation (Figure S10S), while foam cells maintained structural integrity with minor mitochondrial swelling (Figure S10T). Whereas, the CYP1B1‐AS1‐overexpressing group exhibited necrotic endothelial cells featuring chromatin condensation and cellular disintegration (Figure S10U), with profoundly abnormal foam cells containing numerous swollen mitochondria and cystic dilation of the rough endoplasmic reticulum (Figure S10V).

### 3.8 Effect and Mechanism of CYP1B1‐AS1 on AS

**FIGURE 5:**
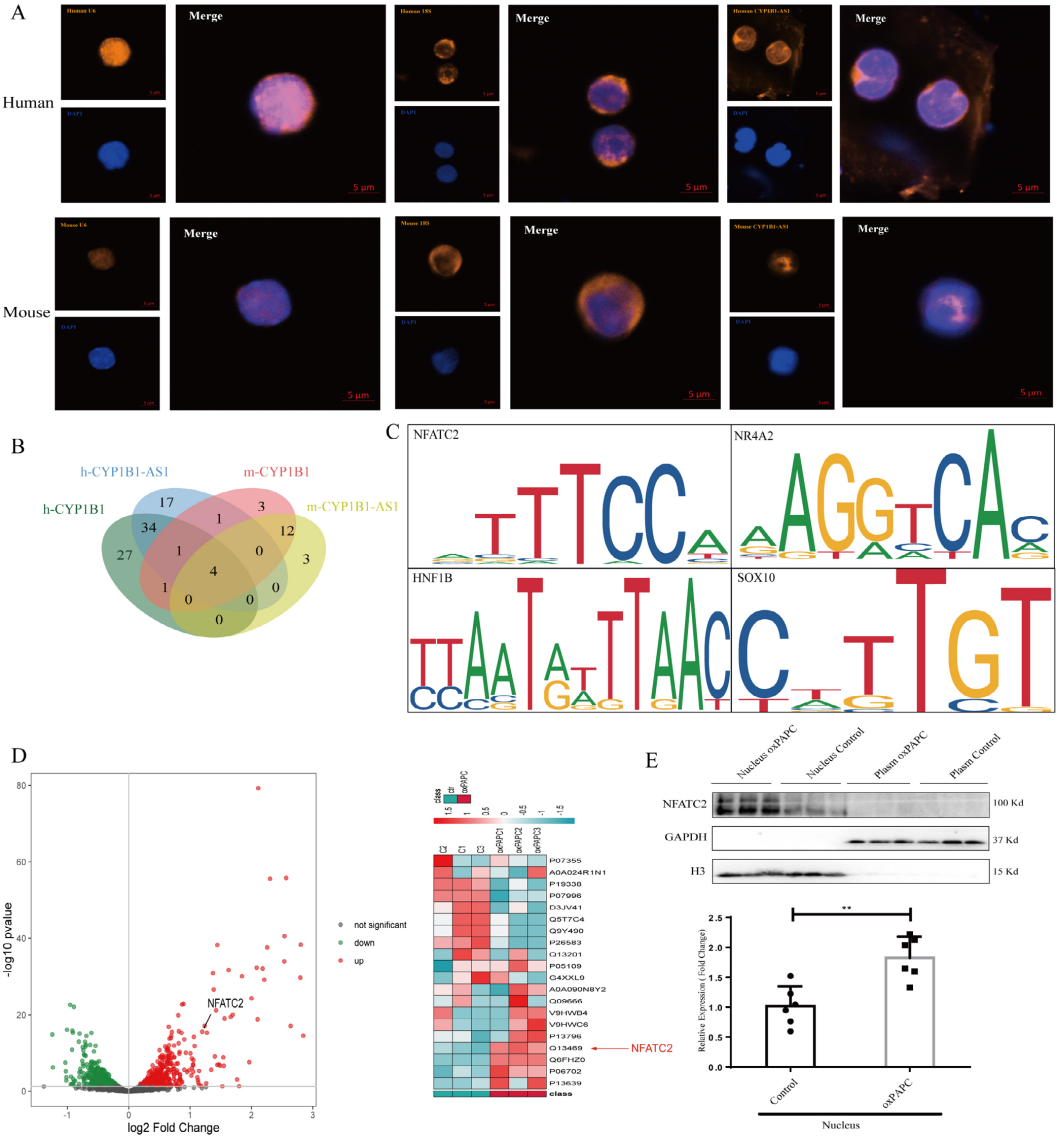
The mechanism of CYP1B1‐AS1 on AS. (A) The subcellular localisation of h‐CYP1B1‐AS1 and m‐CYP1B1‐AS1 by fluorescence in situ hybridization (FISH). (B) The transcription factors binding sites in the promoters of human and mouse CYP1B1 and CYP1B1‐AS1 by JASPAR. (C) The common transcription factor binding motif for human and mouse CYP1B1 and CYP1B1‐AS1. (D, E) The levels of DC nuclear proteins after oxPAPC stimulation by TMT mass spectrometry. (F) The levels of nuclear and cytoplasmic NFATc2 before and after oxPAPC stimulation by Western blot (**p < 0.01).

**FIGURE 6:**
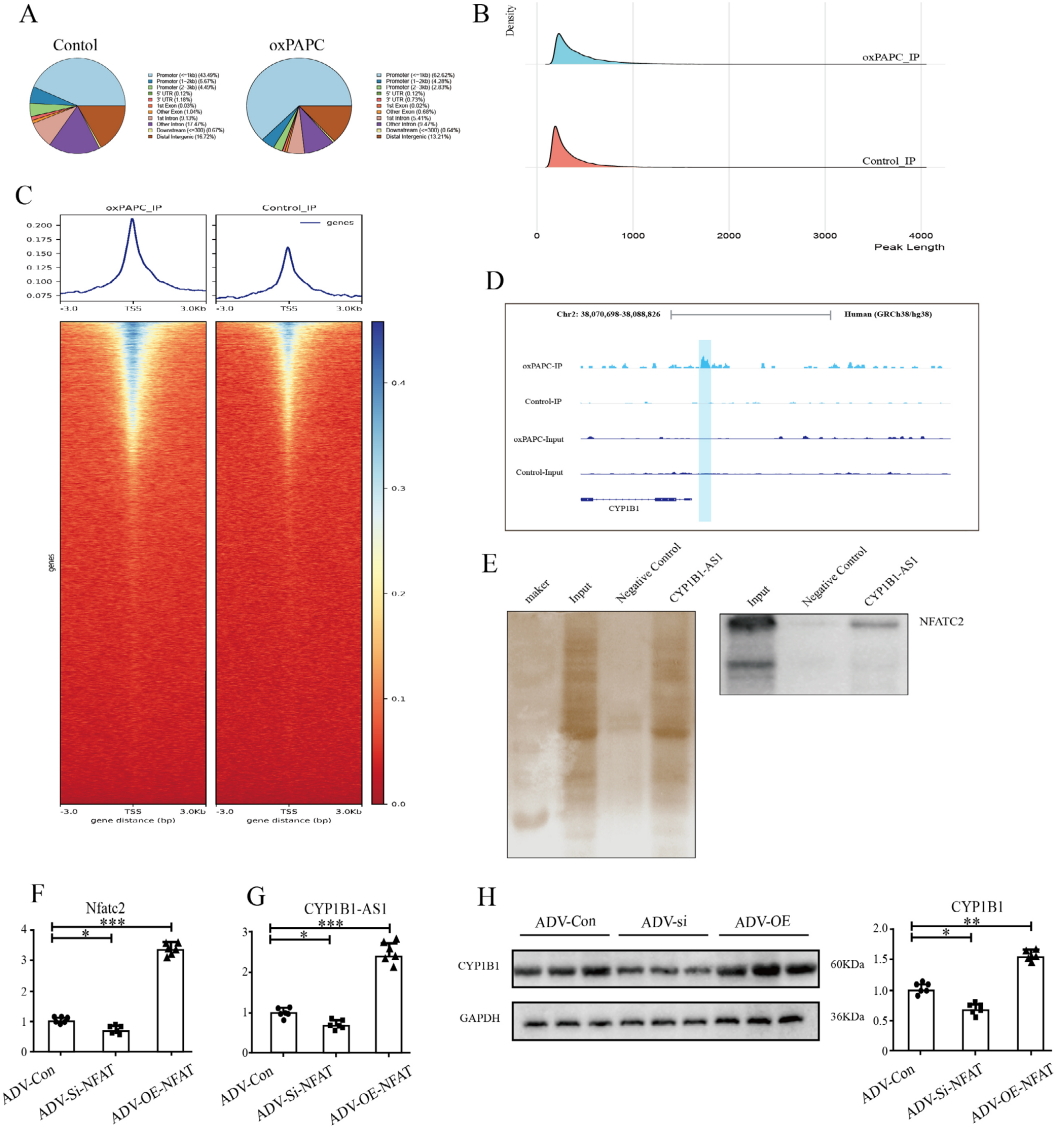
The relationship among oxPAPC, NFATC2, and CYP1B1‐AS1. (A–D) The enrichment of NFATC2 before and after oxPAPC stimulation by ChIP‐Seq. (A) The genomic distribution of enriched peaks. (B) The length distribution of enriched peaks. (C) The distribution of reads around transcription start sites. (D) NFATC2 enrichment in the promoter region sites and sequence information of CYP1B1‐AS1. (E) The binding of CYP1B1‐AS1 to NFATC2 by RNA pulldown assay. (F, G) The expression levels of Nfatc2 and CYP1B1‐AS1 in mouse DCs following Nfatc2 knockdown or overexpression were determined by RT‐PCR. (H) The CYP1B1 expression levels after knockdown and overexpression of Nfatc2 by immunoblotting analysis (*P < 0.05, **P < 0.01, ***P < 0.001).

The subcellular localisation of lncRNAs is closely linked to their functions [42]. Accordingly, we performed fluorescence in situ hybridization (FISH) using U6 and 18S as nuclear and cytoplasmic references, respectively, to determine the localisation of h‐CYP1B1‐AS1 and m‐CYP1B1‐AS1. Both transcripts were predominantly localised to the nucleus (Figure 5A), suggesting their involvement in transcriptional regulation. Given the interspecies homology and antisense relationship between CYP1B1‐AS1 and CYP1B1, we predicted the promoter regulatory sites for mouse CYP1B1, m‐CYP1B1‐AS1, human CYP1B1, and h‐CYP1B1‐AS1 using the JASPAR database (v2022). Screening for binding motifs with JASPAR scores ≥ 8 revealed four common transcription factors (HNF1B, SOX10, NR4A2, NFATC2) across all regulatory regions (Table 6, Figure 5B,C). NFATC2 has emerged as a key candidate because of its role in CD14‐mediated inflammatory responses and DC apoptosis during LPS stimulation [43]. Tandem mass‐tag (TMT) proteomics demonstrated that oxPAPC significantly increased nuclear NFATC2 levels (Figure 5D), as confirmed by western blotting (Figure 5E). To investigate NFATC2's relationship between CYP1B1‐AS1/CYP1B1, we performed ChIP‐seq on oxPAPC‐stimulated DCs. oxPAPC enhanced NFATC2 enrichment at genomic promoters and transcription start sites (TSS) (Figure 6A–C). Differential enrichment analysis using MAnorm2 (threshold: fold change > 2, *p* < 0.01) identified significant NFATC2 binding within the CYP1B1‐AS1 promoter region (Chr2: 38,070,698–38,088,826; strand: +; Figure 6D). Finally, RNA pull‐down assays confirmed the direct binding between lncRNA CYP1B1‐AS1 and NFATC2 (Figure 6E), and NFATC2 overexpression increased the transcriptional levels of CYP1B1‐AS1 (Figure 6F,G) and CYP1B1 (Figure 6H). This suggests a complex mutual recruitment of NFATC2 and CYP1B1‐AS1. In DCs, oxPAPC treatment induced NFATC2 nuclear translocation, where it bound the CYP1B1‐AS1 promoter and increased its transcription. Subsequently, CYP1B1‐AS1 interacted directly with NFATC2, contributing to enhanced AS progression.

**TABLE 6:** JASPAR predicted NFATC2 binding sites and motifs.

Group	Score	Start	End	Strand	Predicted sequence
Human‐CYP1B1	11.35969	1508	1514	−	TTTTCCA
	8.660004	1803	1809	−	GTTTCCA
	8.593737	20	26	+	TCTTCCA
	8.134328	1129	1135	+	CTTTCCA
Human‐CYP1B1‐AS1	9.490151	24	30	−	TTTTCCT
	9.186889	209	215	+	TTTTCCC
	8.660004	547	553	+	GTTTCCA
	8.593737	976	982	−	TGTTCCA
	8.276758	1180	1186	+	TTTTCCG
	8.276758	1612	1618	+	TTTTCCG
	8.276758	1741	1747	−	TTTTCCG
	8.068061	1106	1112	+	TATTCCA
Mouse‐CYP1B1	11.35969	762	768	+	TTTTCCA
	9.490151	1556	1562	+	TTTTCCT
	9.490151	1763	1769	−	TTTTCCT
	9.044458	1663	1669	−	ATTTCCA
	8.660004	1422	1428	+	GTTTCCA
Mouse‐CYP1B1‐AS1	11.35969	889	895	+	TTTTCCA
	9.490151	184	190	−	TTTTCCT
	9.490151	1534	1540	−	TTTTCCT
	8.134328	1304	1310	−	CTTTCCA
	8.134328	1705	1711	+	CTTTCCA

## 4 Discussion

The innate immune system detects tissue injury through pattern recognition receptors, promoting the activation and development of adaptive immunity. DCs serve as pivotal bridging cells between adaptive and innate immunity and recognise specific DAMPs to initiate immune responses [2, 8]. These receptors couple tissue damage with downstream signalling transduction in immune cells, upregulating the expression of multiple inflammatory mediators. Given their close association with the upstream mechanisms of AS progression, we selected DCs for our research.

DCs serve as the cellular bridge linking the “damage‐response” axis in AS, while oxPAPC functions as its molecular mediator. This premise is supported by three key observations. First, oxPAPC, a primary bioactive component of ox‐LDL, originates from tissue injury sites. Second, oxPAPC specifically induces DC rather than macrophage hyperactivation. Furthermore, oxPAPC triggers gene cluster alterations in mice that mirror those observed in patients with proatherogenic lipid profiles. Ultimately, our previous study identified differential expression of DC‐derived lncRNAs during acute coronary syndrome (ACS) [44], a critical stage of AS. Notably, the current study detected elevated serum oxPAPC levels in patients with ACS. These findings motivated the present investigation into oxPAPC‐induced transcriptomic changes in DCs.

Recent studies have identified multiple lncRNAs that regulate DC phenotype and AS progression. However, the lncRNA transcriptome of oxPAPC‐induced hyperactivated DCs remains unclear. Consequently, we focused on how oxPAPC‐driven lncRNA alterations in DCs influence AS pathogenesis. Given the prolonged and heterogeneous pathophysiological progression of AS, we hypothesised that specific lncRNAs' expression requires specific oxPAPC concentrations or synergistic stimulation by ox‐LDL components. To address this, we profiled transcriptomic responses across multiple concentrations of ox‐LDL and oxPAPC + ox‐LDL cotreatments. Notably, the CYP1B1‐AS1 transcript was consistently upregulated at all tested concentrations, with a positive correlation with oxPAPC dosage. This robust and dose‐dependent response prompted us to focus on CYP1B1‐AS1, suggesting that CYP1B1‐AS1 is involved in the entire spectrum of AS progression.

LncRNA conservation across species is generally low. For instance, human CYP1B1‐AS1 (1776 bp) shares minimal sequence homology with its murine counterpart Gm33055 (432 bp), as identified via UCSC LiftOver. Assessing functional conservation, particularly conservation of the promoter region, is of paramount importance. RBP map analysis revealed > 85% overlap in transcription factors regulating the CYP1B1‐AS1 and Gm33055 promoters, establishing Gm33055 as the functional murine ortholog of CYP1B1‐AS1. Given that antisense lncRNAs often regulate adjacent transcripts, we calculated the Pearson correlation coefficient between CYP1B1‐AS1 and CYP1B1 (*r* = 0.97), which strongly supported a regulatory interplay. WGCNA indicated that the CYP1B1‐co‐expressed genes are involved in transcriptional regulation and immune‐inflammatory pathways. The abundance of co‐expressed transcription factors suggests that CYP1B1 operates upstream of oxPAPC‐induced DC hyperactivation. Subsequent scRNA‐seq analysis of human AS lesions (GEO database) confirmed CYP1B1 association with AS. Although both DCs and macrophages overexpressed CYP1B1 in AS, DCs exhibited a disproportionately higher intensity (Figure S3E). CellChat analysis further revealed more robust chemotactic and inflammatory interactions between CYP1B1^+^ DCs and proliferating T cells than CYP1B1^+^ macrophages, underscoring the pivotal role of CYP1B1^+^ DCs in AS progression.

Although oxPAPC‐mediated CD14 internalisation has been reported to block TLR4 signalling, we propose an alternative model contingent on CD14 availability. First, TLR4 blockade requires membrane CD14; however, oxPAPC itself depletes CD14 via internalisation. Second, in CD14‐deficient cells, oxPAPC enters via endocytosis, binds to MD2, and activates TLR4 signalling. Third, this reconciles the paradox of oxPAPC (a major ox‐LDL component) acting as a TLR4 antagonist, despite the well‐established TLR4‐activating role of ox‐LDL in AS intimal inflammation. Forth, oxPAPC‐induced DC hyperactivation sustains IL‐1β release without pyroptosis. Given elevated IL‐1β in ACS patients and its role in promoting vulnerable plaque rupture (often containing dead foam cells/MΦs), persistent IL‐1β accumulation may ultimately overwhelm the anti‐pyroptotic effect, triggering DC death and uncontrolled inflammation. Therefore, we hypothesised that sustained oxPAPC stimulation following CD14 depletion may activate the TLR4 pathway. Our data confirmed that progressive CD14 depletion enables oxPAPC‐mediated TLR4 activation, consistent with chronic AS intimal inflammation and established TLR4 involvement in AS progression.

CD11b^+^ DCs (cDC2 subset) preferentially activate CD4^+^ T cells via MHC‐II, contrasting with cDC1 cross‐presentation to CD8^+^ T cells. The scRNA‐seq and CellChat analyses of AS plaques aligned with this paradigm. Since CD4^+^ T cells dominate AS progression, we investigated the ApoB antigen‐presenting capacity of DCs and their ability to stimulate lymphocyte proliferation. Although the NCBI data indicated predominantly hepatic ApoB expression, western blotting revealed elevated ApoB levels in oxPAPC‐stimulated DCs. Whether this reflects de novo expression or enhanced phagocytosis remains unclear, regardless of whether it indicates increased potential for ApoB presentation. Lymphocyte proliferation assays confirmed the enhanced T cell‐stimulatory capacity of oxPAPC‐treated DCs.

CYP1B1, a heme thiolate monooxygenase implicated in diabetes, hypertension, AS, and cancer, is an AhR‐induced transcript. The link between AhR and oxPAPC‐induced DC hyperactivation is unexplored, although TLR activation upregulates AhR, which amplifies TLR‐induced IL‐1β release in DCs. Sustained oxPAPC stimulation promotes DC death, elevated co‐stimulatory molecule expression, and enhanced lymphocyte activation, consistent with known AS features: pyroptosis contributing to plaque instability, lipid‐rich plaque vulnerability, and elevated circulating IL‐1β in ACS. This finding also clarifies whether persistent IL‐1β accumulation counteracts the anti‐pyroptotic effect of oxPAPC.

GEO data suggest that CYP1B1‐high DCs accelerate the panvascular nature of AS, oxPAPC‐induced DC migratory capacity, C57BL/6J genetic homogeneity, and the clinical relevance of adoptive immunotherapy; hence, we employed the adoptive transfer of ADV‐DC‐OE to investigate DC‐derived CYP1B1‐AS1. This approach confirmed that the DC‐derived lncRNA CYP1B1‐AS1 accelerates AS progression.

FISH localised CYP1B1‐AS1 to the nucleus, indicating its transcriptional regulation. Although RBP map predicted > 85% similarity in regulatory proteins for the m‐CYP1B1‐AS1 and h‐CYP1B1‐AS1 promoters, we identified conserved transcription factor‐binding motifs (NFATC2, SOX10, NR4A2, and HNF1B) in human and mouse CYP1B1‐AS1/CYP1B1 promoters. NFATC2 was prioritised because (1) CD14‐dependent activation by LPS (a structural analog of oxPAPC) modulates DC viability and (2) western blotting and TMT proteomics confirmed oxPAPC‐induced nuclear NFATC2 accumulation. NFATC2‐driven CYP1B1‐AS1 induction resembles the AhR‐CYP1B1‐AhR positive feedback loop. To our knowledge, our findings provide the first evidence of NFATC2 involvement in CYP1B1 transcriptional regulation.

This study had some limitations. First, NFATC2 activation depends on CD14‐ and TLR4‐mediated nuclear translocation with crosstalk between the TLR4 and AhR‐CYP1B1 pathways. Lacking TLR4‐knockout models precluded further investigation of oxPAPC‐induced CYP1B1‐AS1 signalling; however, this does not invalidate the core finding of NFATC2 involvement in CYP1B1‐AS1/CYP1B1 regulation. Second, although we demonstrated AS acceleration by CYP1B1‐overexpressing DCs, experimental constraints prevented elucidating the precise mechanisms (e.g., IL‐1β secretion vs. T cell activation post‐lymphoid homing).

In conclusion, this study establishes that DC‐derived lncRNA CYP1B1‐AS1 is functionally conserved, transcriptionally regulated by NFATC2, and central to oxPAPC‐induced DC hyperactivation. Sustained oxPAPC stimulation promotes CD11b^+^ CD11c^+^ DC differentiation, enhances antigen presentation, and amplifies CYP1B1‐AS1/CYP1B1 regulatory circuit, collectively driving AS progression. These findings highlight a novel molecular pathway linking innate immune sensing to adaptive immune activation in atherogenesis.

## Author Contributions

**Yuheng Cheng:** writing – original draft, project administration, methodology, investigation, formal analysis, data curation. **Lang Ni, Changhao Ke, Yuanjie He, Youyang Huang, Shiwan Lu:** investigation, data curation. **Yongchao Zhao:** software, formal analysis, data curation. **Junbo Ge, Bei Shi:** writing – review and editing, supervision. **Zhenglong Wang:** writing – review and editing, supervision, conceptualization, methodology.

## Funding

This work was funded by The Regional Fund Project of the National Natural Science Foundation of China (project name: Differential expression of DC‐derived lncRNAs in acute coronary syndrome and their regulatory mechanism; project number: 81760072). Guizhou Provincial Science and Technology Foundation Key Project (name: Effects and mechanisms of oxPAPC‐induced DC‐derived lncRNA on AS; number: ZK [2024] Key 085).

## Ethics Statement

The clinical research conducted in this study was approved by the Ethics Committee of the Affiliated Hospital of Zunyi Medical University (Ethical Approval Number: KLLY‐2021‐099). All participants were fully informed of the study procedures and provided written informed consent before participation. Animal experiments were performed in strict compliance with the National Institutes of Health (NIH) Guide for the Care and Use of Laboratory Animals and adhered to the ethical standards established by the Institutional Animal Care and Use Committee of Zunyi Medical University. All experimental protocols involving animals were conducted in accordance with the institutional guidelines to ensure humane treatment and welfare throughout the study period.

## Consent

All participants included in this study provided informed consent for the publication of the results and accompanying data.

## Conflicts of Interest

The authors declare no conflicts of interest.

## Supporting information


**Figure S1:** The assessment of conservative and non‐coding properties for Gm33055. (A) The homology of the upstream promoter of CYP1B1‐AS1 among different species by UCSC comparative genomic track. (B) The successful introduction of the T7 promoter by First‐generation sequencing confirmed. (C, D) The results of Coomassie Brilliant Blue staining and transcription‐translation in vitro. (E) The construction of the T7‐Gm33055 plasmid.

**Figure S2:** WGCNA analysis of genes co‐expressed with CYP1B1. (A) Clustering dendrogram of different samples. (B) Correlation between different modules. (C) Clustering dendrogram of the cyan module. (D) Correlation between modules at different soft thresholding values. (E) Heatmap of the TOM matrix in the CYP1B1 co‐expression network. (F) Co‐expressed genes of CYP1B1 under the cyan module.

**Figure S3:** Single‐cell analysis of atherosclerosis based on the GEO database. (A) Marker genes of different cell type (T_cells: CD3D, CD3E, CD3G; CD4_T: CD4; CD8_T: CD8A, CD8B; γδ_T: TRDC, TRGC1; B_cells: CD19, CD79A, CD79B; pDC: LILRA4, IL3RA; moDC: ITGAX, CD14, SIRPA; cDC2: CD1C, FCER1A, Macrophages: CD68, FCGR3A, CD163, MRC1, MSR1; Mast_Cells: MS4A2, KIT, TPSAB1). (B–D) Cell composition of different samples. (B: Control, C: Calcified core, D: Atheromatous plaque). (E) CYP1B1 expression levels in different cell types across various samples. (F) Calcified Atherosclerosis Core vs. Control: CYP1B1 expression across different cell type (Coloured point: differentially expressed genes, log_2_FoldChange > 0.25 and adjusted *p*‐value < 0.01).

**Figure S4:** (A) CellChat assessment of changes in intercellular communication: Blue indicates an increase; Red indicates a decrease. (B) CellChat assessment of changes in the strength of intercellular communication: Blue indicates an increase; Red indicates a decrease. (C, D) Heatmap of intercellular communication numbers and strength (C: Control vs. Atherosclerosis, D: Control vs. Calcified Atherosclerosis Core).

**Figure S5:** Heatmap of incoming signal patterns for Control/Atherosclerosis/Calcified Core.

**Figure S6:** Heatmap of outgoing signal patterns for Control/Atherosclerosis/Calcified Core.

**Figure S7:** Pattern of incoming‐interaction strength vs. outgoing‐interaction strength for different cell types in Control, Atherosclerosis, and Calcified Core.

**Figure S8:** (A) RPKM levels of APOB across different tissues sourced from the NCBI database. (B) ApoB relative expression levels in mouse DC under different stimulation conditions (*p < 0.05, **p < 0.01, ***p < 0.001, ****p < 0.0001).

**Figure S9:** (A, B) ELISA measurement of relative IL‐1β and oxPAPC levels at different time points of myocardial infarction and AS mice. (C) Flow cytometry analysis of the ratio of CD11b^+^CD11c^+^ moDC in the peripheral blood of mice at different time points following myocardial infarction. (D) Flow cytometry analysis of CD80 and CD86 expression levels on moDCs in the peripheral blood of mice at different time points following myocardial infarction (with LPS‐stimulated mouse DCs as a positive control) (*p < 0.05, **p < 0.01, ***p < 0.001, ****p < 0.0001).

**Figure S10:** (A–L) Tissue staining of aortic sinus and aorta in *ApoE* ^−/−^mice treated with ADV‐CON or ADV‐OE‐CYP1B1‐AS1. (A) HE staining of aortic sinus (ADV‐CON). (B) Masson staining of aortic sinus (ADV‐CON). (C) Oil Red O staining of aortic sinus (ADV‐CON). (D) HE staining of aorta (ADV‐CON). (E) Masson staining of aorta (ADV‐CON). (F) Oil Red O staining of aorta (ADV‐CON). (G) HE staining of aortic sinus and aorta (ADV‐OE‐CYP1B1‐AS1). (H) Masson staining of aortic sinus (ADV‐OE‐CYP1B1‐AS1). (I) Oil Red O staining of aortic sinus (ADV‐OE‐CYP1B1‐AS1). (J) HE staining of aorta (ADV‐OE‐CYP1B1‐AS1). (K) Masson staining of aorta (ADV‐OE‐CYP1B1‐AS1). (L) Oil Red O staining of aorta (ADV‐OE‐CYP1B1‐AS1). (M–R) Quantitative analysis of plaque composition. (M, P) Relative plaque area in aortic sinus (M) and aorta (P) (ADV‐CON vs. ADV‐OE‐CYP1B1‐AS1). (N, Q) Relative plaque fibrosis in aortic sinus (N) and aorta (Q) (ADV‐CON vs. ADV‐OE‐CYP1B1‐AS1). (O, R) Relative plaque lipid content in aortic sinus (O) and aorta (R) (ADV‐CON vs. ADV‐OE‐CYP1B1‐AS1). (S–V) Transmission electron microscopy (TEM) of aortic sinus. (S, T) Representative TEM images (ADV‐CON). (U, V) Representative TEM images (ADV‐OE‐CYP1B1‐AS1) (*p* < 0.05).

## Data Availability

Research data associated with this investigation are accessible from the corresponding author following appropriate scientific justification.
