# Supplementary figures and images for: oxPAPC‐Mediated lncRNA CYP1B1‐AS1 From Dendritic Cells Accelerates Atherosclerosis
Source: J Cell Mol Med. 2026 Feb 27;30(5):e71066. doi: 10.1111/jcmm.71066 (PMC12948649; doi:10.1111/jcmm.71066)

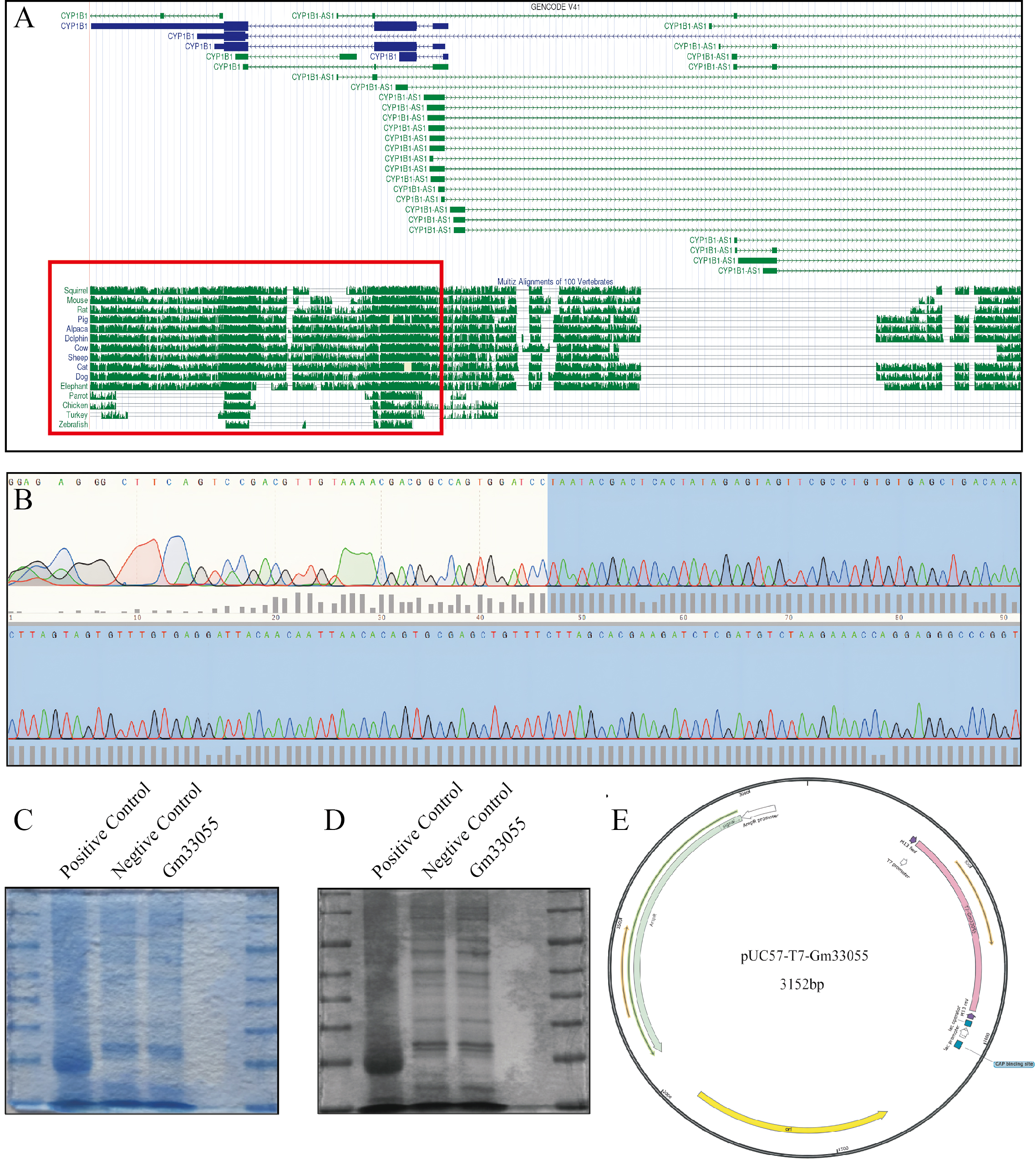

Supplement: Supplementary file 1 — Figure S1: The assessment of conservative and non‐coding properties for Gm33055. (A) The homology of the upstream promoter of CYP1B1‐AS1 among different species by UCSC comparative genomic track. (B) The successful introduction of the T7 promoter by First‐generation sequencing confirmed. (C, D) The results of Coomassie Brilliant Blue staining and transcription‐translation in vitro. (E) The construction of the T7‐Gm33055 plasmid. [file JCMM-30-e71066-s006.jpg]

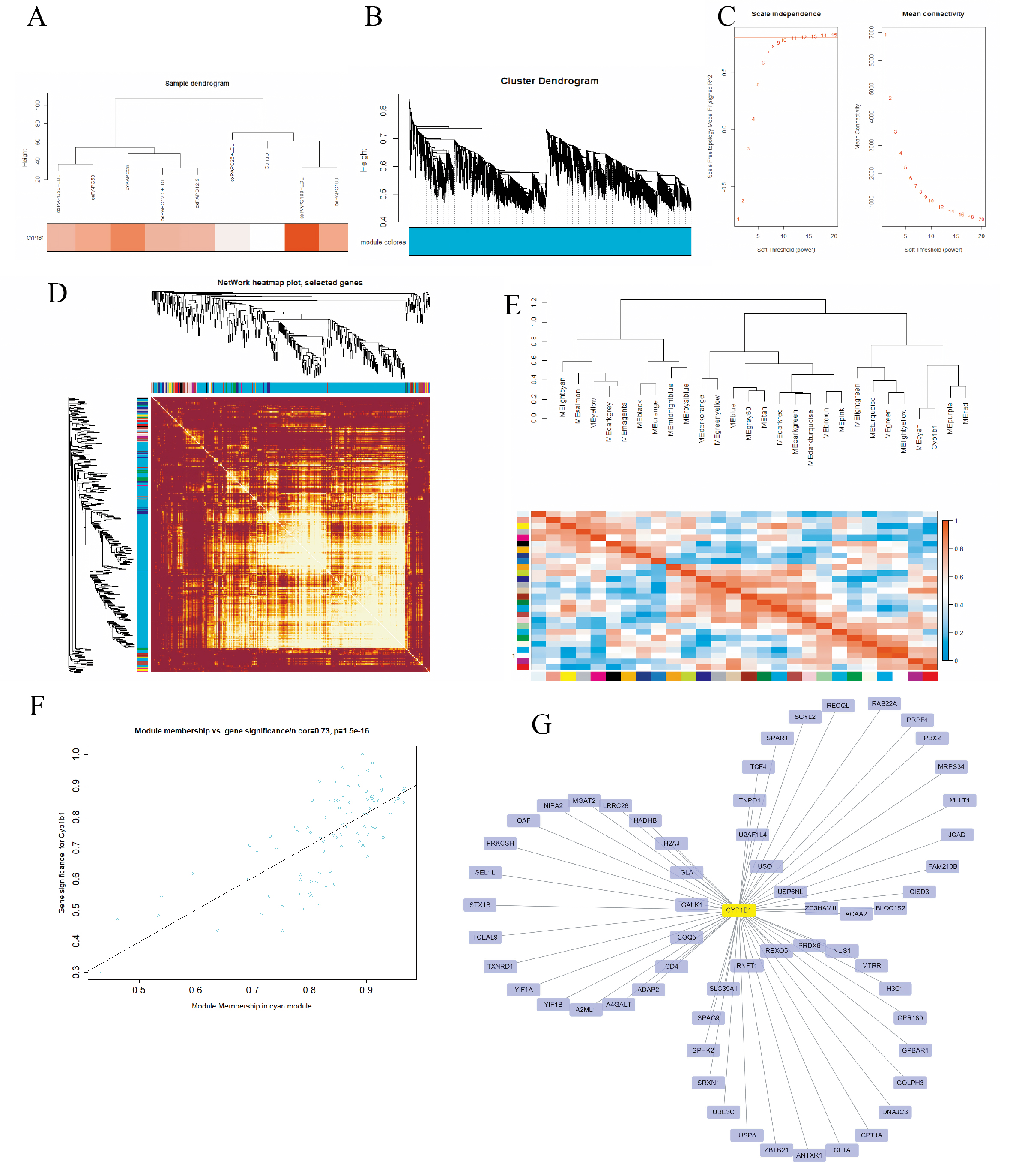

Supplement: Supplementary file 2 — Figure S2: WGCNA analysis of genes co‐expressed with CYP1B1. (A) Clustering dendrogram of different samples. (B) Correlation between different modules. (C) Clustering dendrogram of the cyan module. (D) Correlation between modules at different soft thresholding values. (E) Heatmap of the TOM matrix in the CYP1B1 co‐expression network. (F) Co‐expressed genes of CYP1B1 under the cyan module. [file JCMM-30-e71066-s007.jpg]

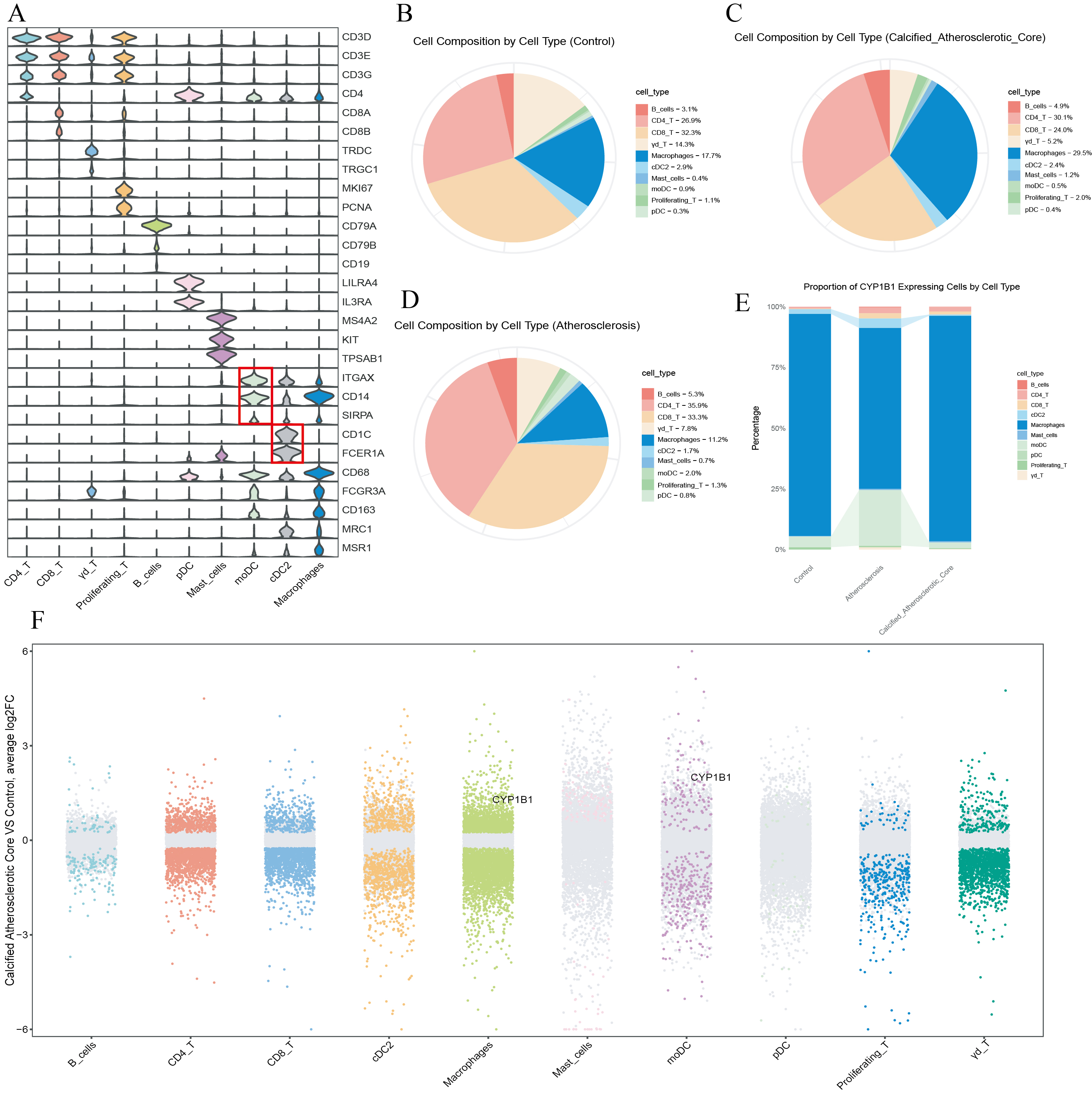

Supplement: Supplementary file 3 — Figure S3: Single‐cell analysis of atherosclerosis based on the GEO database. (A) Marker genes of different cell type (T_cells: CD3D, CD3E, CD3G; CD4_T: CD4; CD8_T: CD8A, CD8B; γδ_T: TRDC, TRGC1; B_cells: CD19, CD79A, CD79B; pDC: LILRA4, IL3RA; moDC: ITGAX, CD14, SIRPA; cDC2: CD1C, FCER1A, Macrophages: CD68, FCGR3A, CD163, MRC1, MSR1; Mast_Cells: MS4A2, KIT, TPSAB1). (B–D) Cell composition of different samples. (B: Control, C: Calcified core, D: Atheromatous plaque). (E) CYP1B1 expression levels in different cell types across various samples. (F) Calcified Atherosclerosis Core vs. Control: CYP1B1 expression across different cell type (Coloured point: differentially expressed genes, log2FoldChange > 0.25 and adjusted p‐value < 0.01). [file JCMM-30-e71066-s002.jpg]

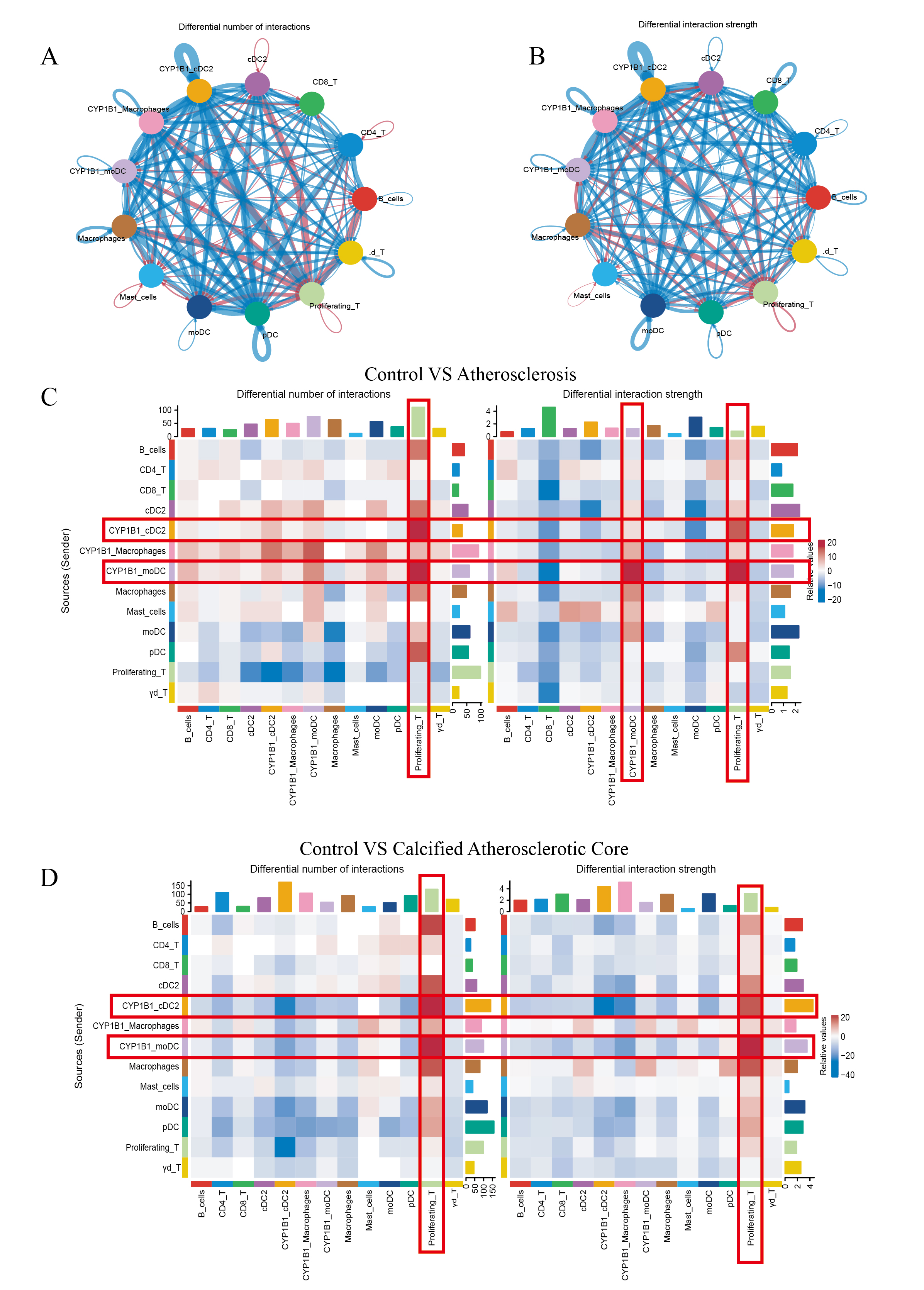

Supplement: Supplementary file 4 — Figure S4: (A) CellChat assessment of changes in intercellular communication: Blue indicates an increase; Red indicates a decrease. (B) CellChat assessment of changes in the strength of intercellular communication: Blue indicates an increase; Red indicates a decrease. (C, D) Heatmap of intercellular communication numbers and strength (C: Control vs. Atherosclerosis, D: Control vs. Calcified Atherosclerosis Core). [file JCMM-30-e71066-s008.jpg]

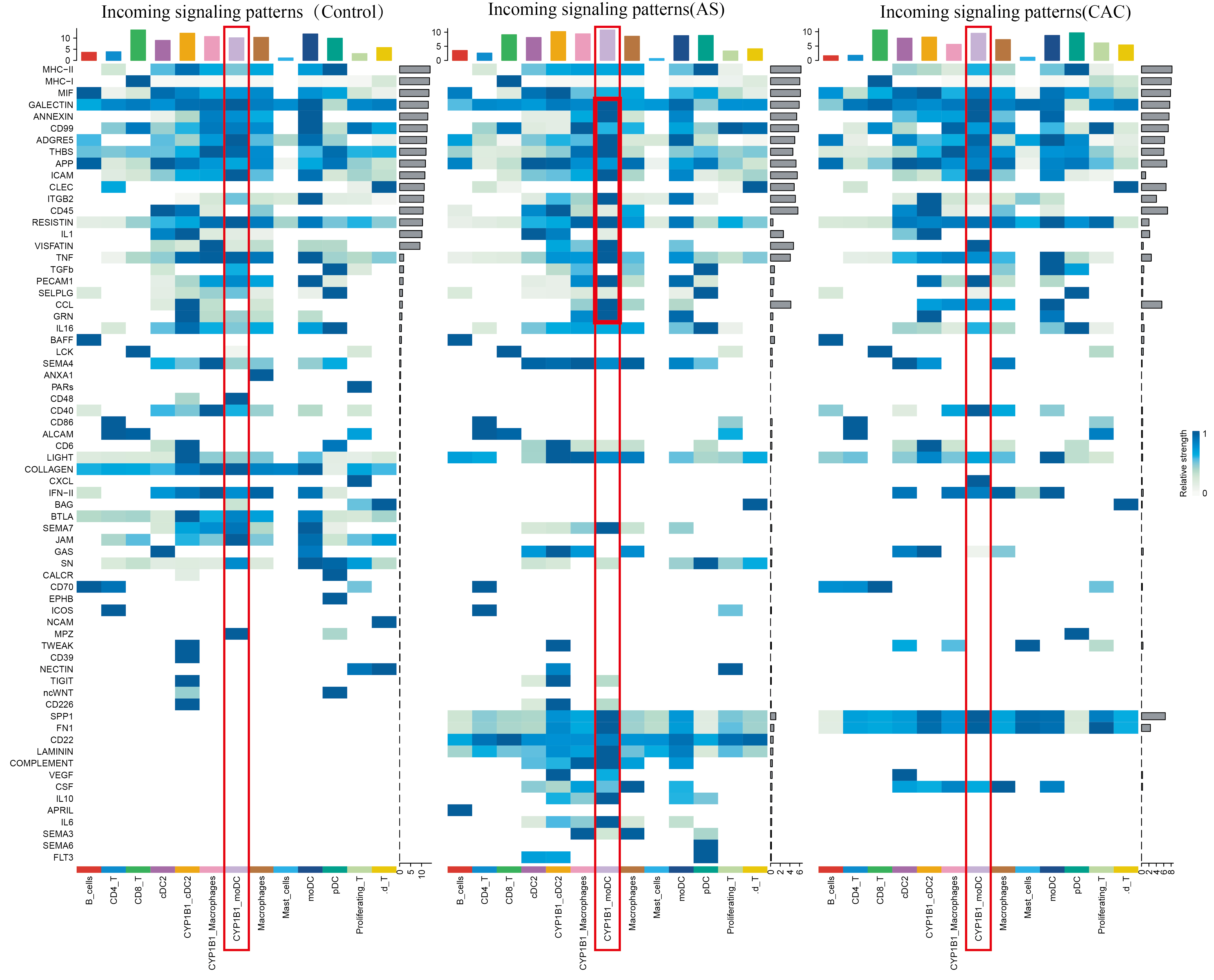

Supplement: Supplementary file 5 — Figure S5: Heatmap of incoming signal patterns for Control/Atherosclerosis/Calcified Core. [file JCMM-30-e71066-s004.jpg]

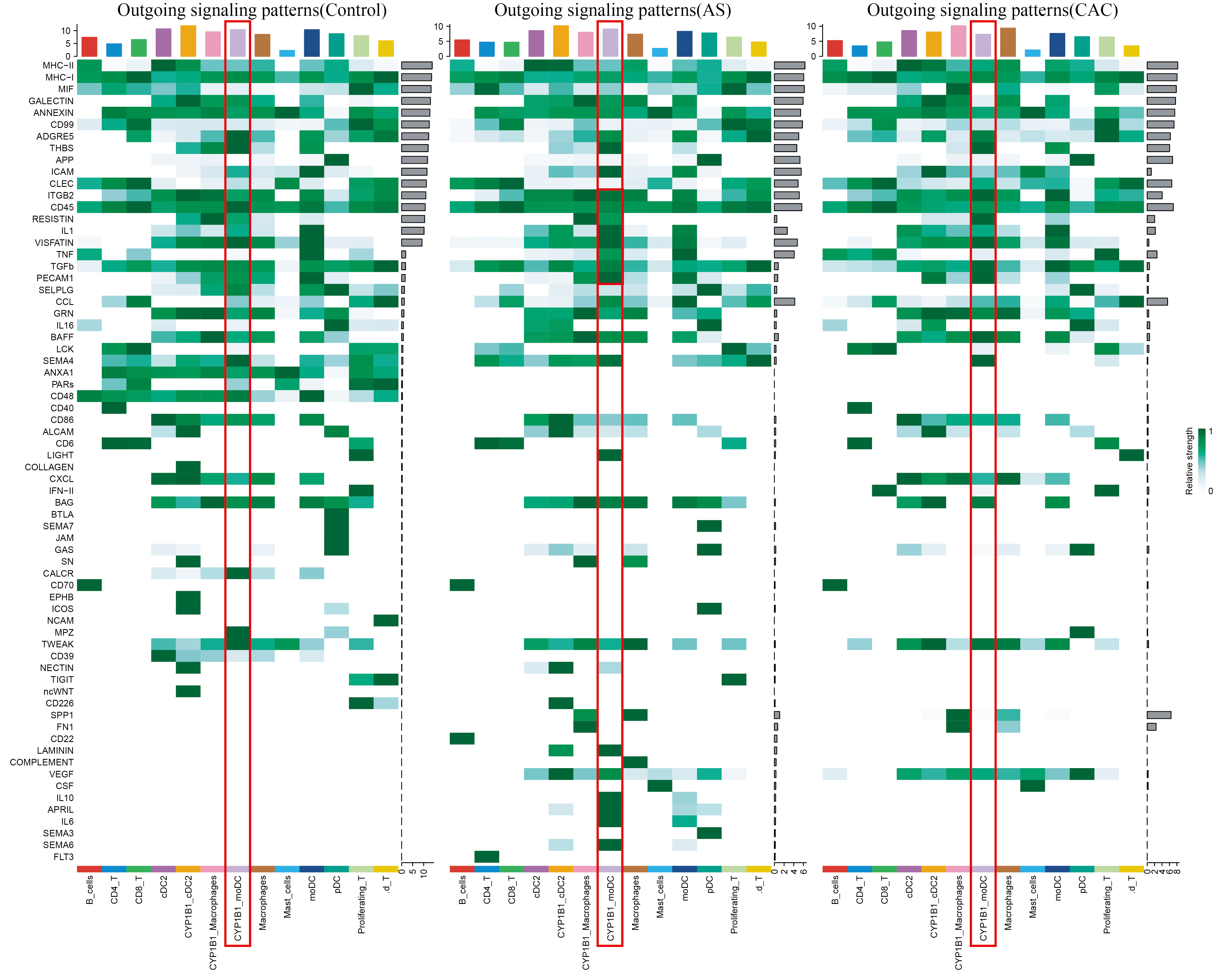

Supplement: Supplementary file 6 — Figure S6: Heatmap of outgoing signal patterns for Control/Atherosclerosis/Calcified Core. [file JCMM-30-e71066-s003.jpg]

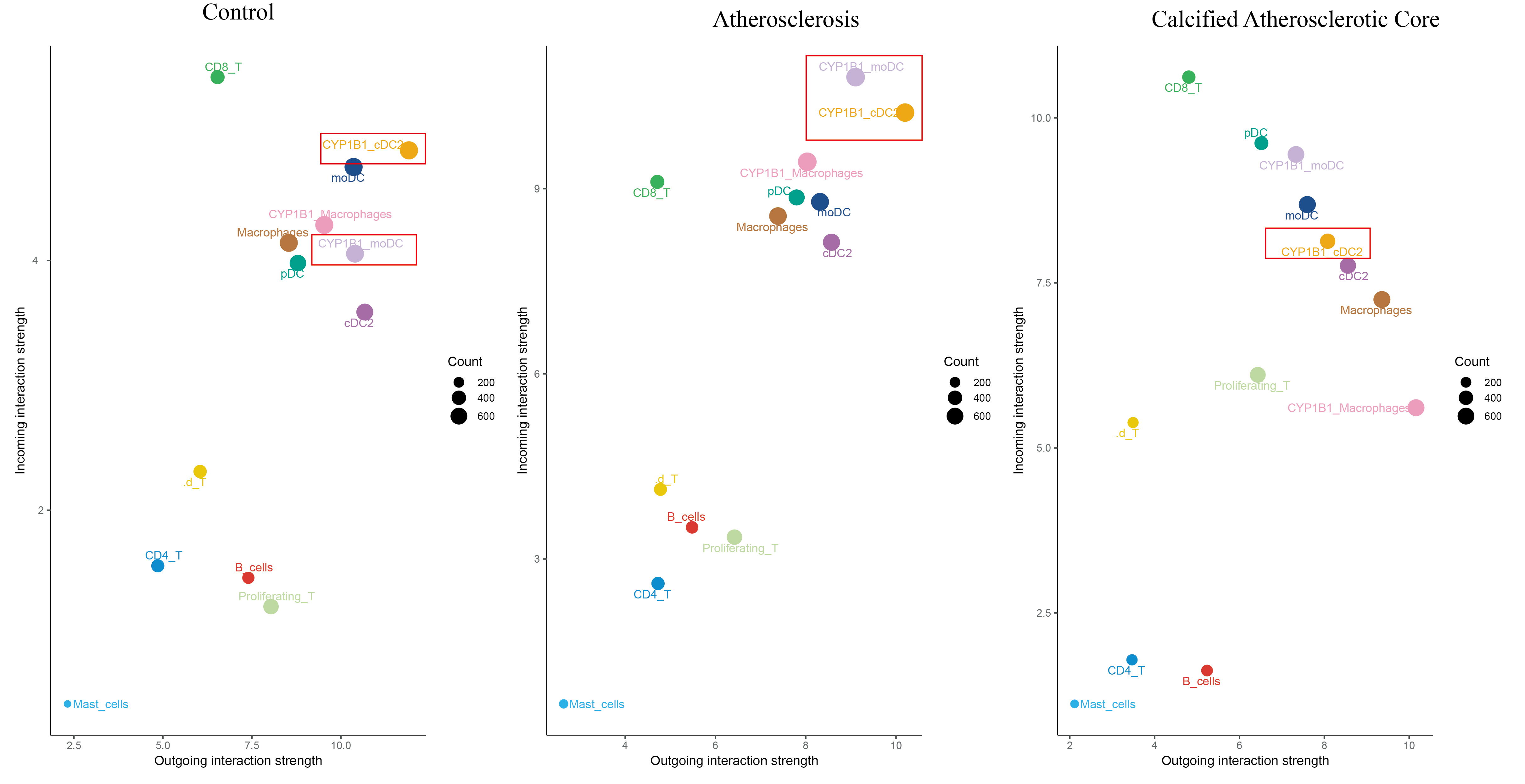

Supplement: Supplementary file 7 — Figure S7: Pattern of incoming‐interaction strength vs. outgoing‐interaction strength for different cell types in Control, Atherosclerosis, and Calcified Core. [file JCMM-30-e71066-s009.jpg]

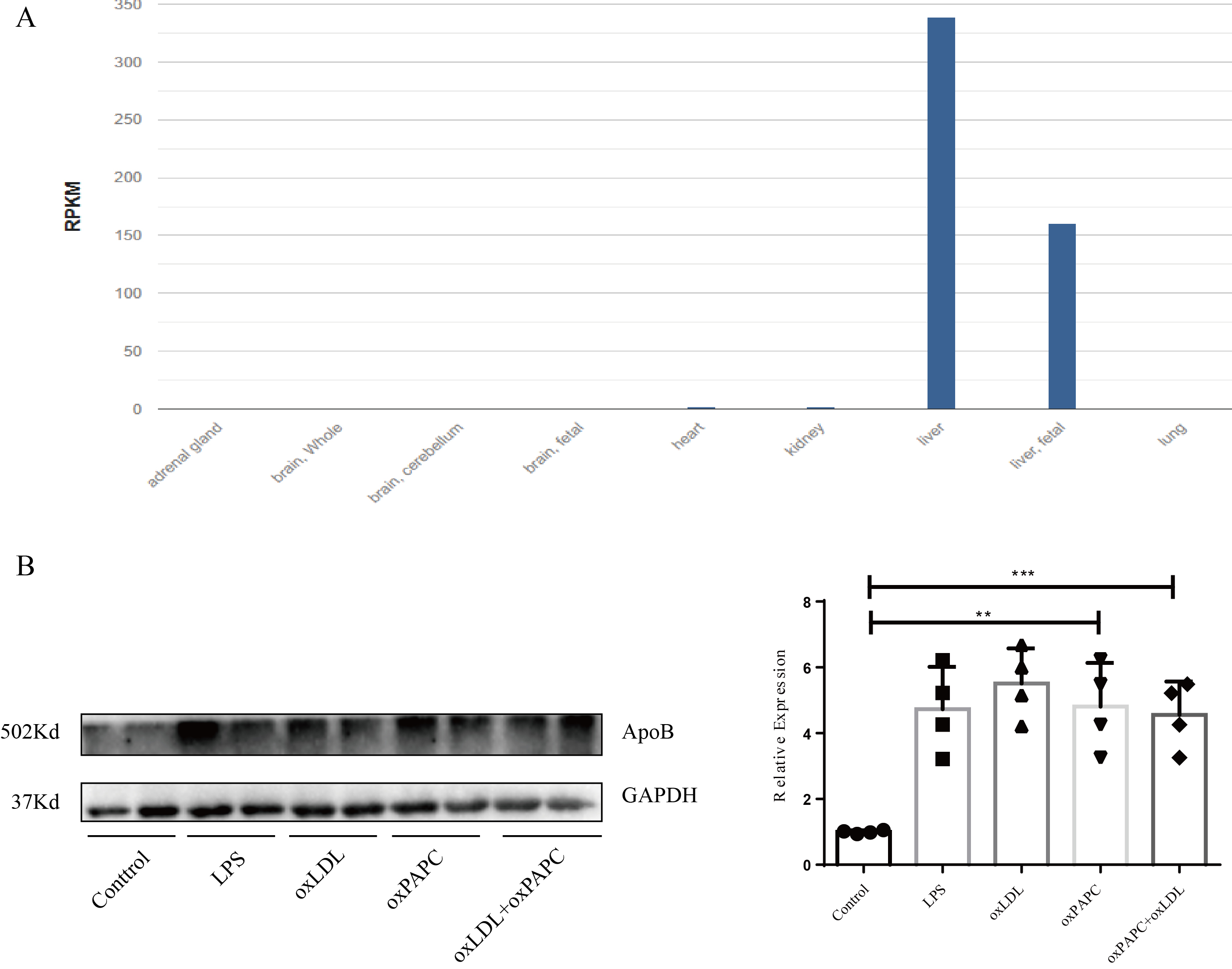

Supplement: Supplementary file 8 — Figure S8: (A) RPKM levels of APOB across different tissues sourced from the NCBI database. (B) ApoB relative expression levels in mouse DC under different stimulation conditions (*p < 0.05, **p < 0.01, ***p < 0.001, ****p < 0.0001). [file JCMM-30-e71066-s001.jpg]

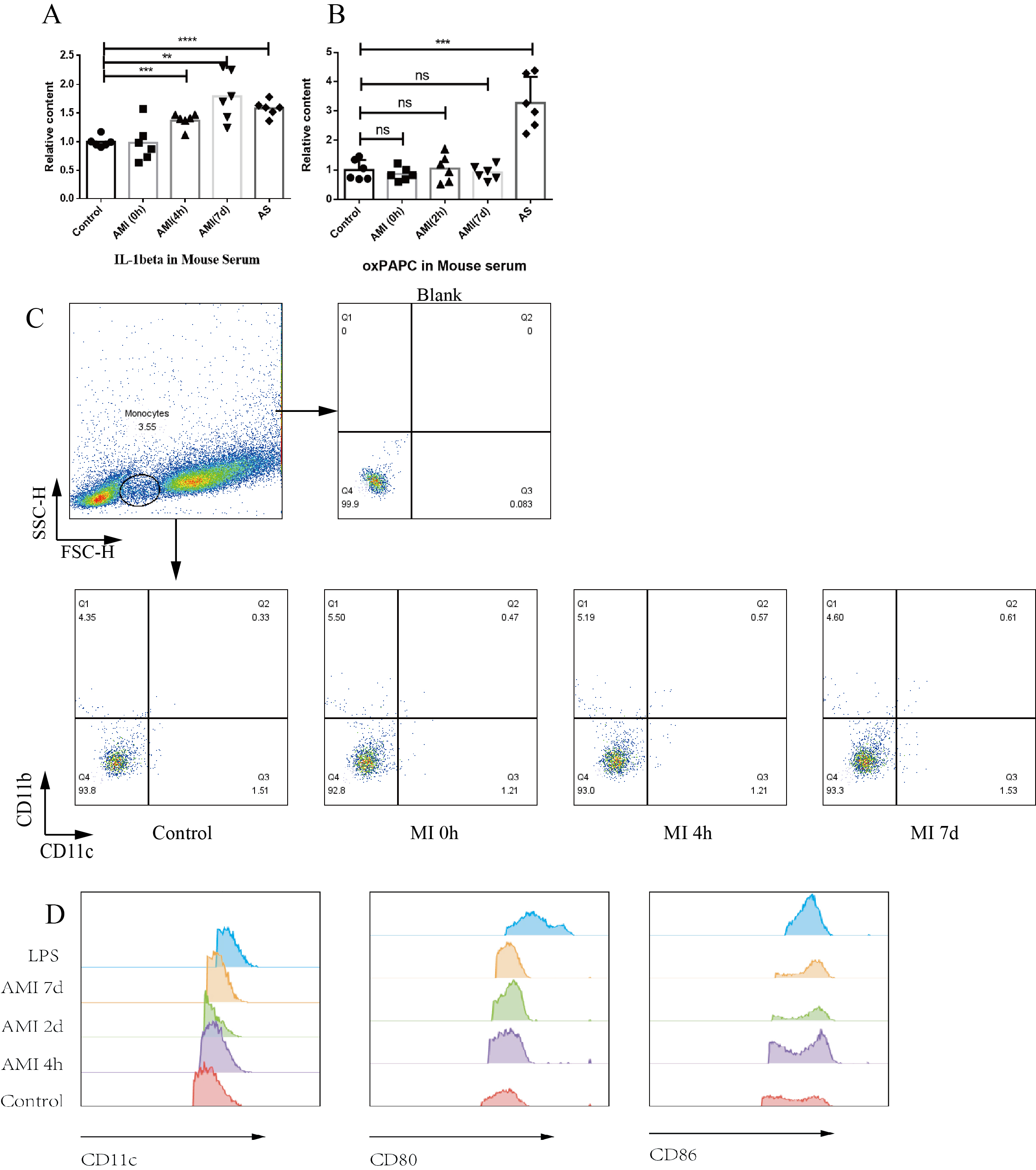

Supplement: Supplementary file 9 — Figure S9: (A, B) ELISA measurement of relative IL‐1β and oxPAPC levels at different time points of myocardial infarction and AS mice. (C) Flow cytometry analysis of the ratio of CD11b+CD11c+ moDC in the peripheral blood of mice at different time points following myocardial infarction. (D) Flow cytometry analysis of CD80 and CD86 expression levels on moDCs in the peripheral blood of mice at different time points following myocardial infarction (with LPS‐stimulated mouse DCs as a positive control) (*p < 0.05, **p < 0.01, ***p < 0.001, ****p < 0.0001). [file JCMM-30-e71066-s005.jpg]

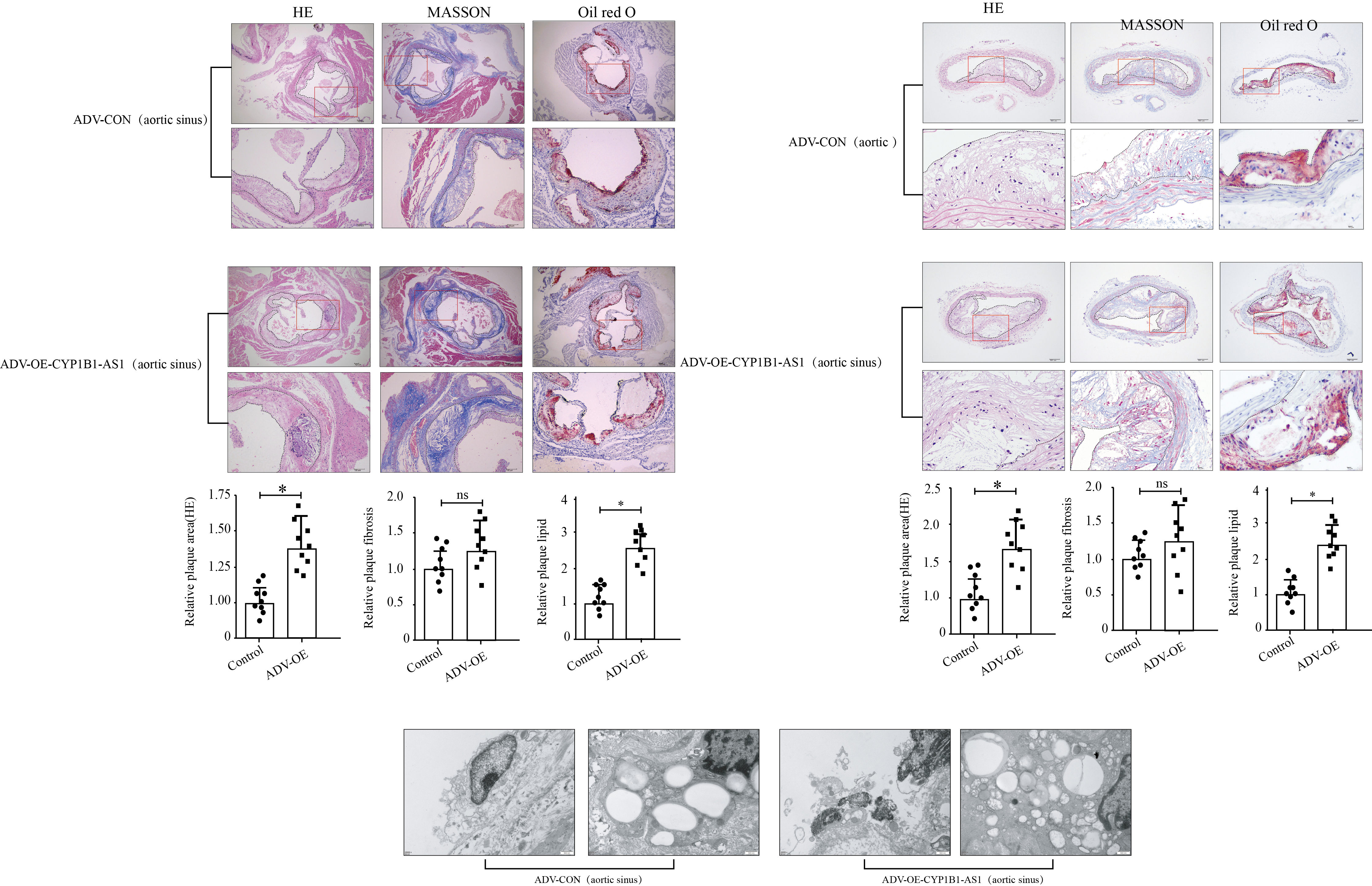

Supplement: Supplementary file 10 — Figure S10: (A–L) Tissue staining of aortic sinus and aorta in ApoE −/−mice treated with ADV‐CON or ADV‐OE‐CYP1B1‐AS1. (A) HE staining of aortic sinus (ADV‐CON). (B) Masson staining of aortic sinus (ADV‐CON). (C) Oil Red O staining of aortic sinus (ADV‐CON). (D) HE staining of aorta (ADV‐CON). (E) Masson staining of aorta (ADV‐CON). (F) Oil Red O staining of aorta (ADV‐CON). (G) HE staining of aortic sinus and aorta (ADV‐OE‐CYP1B1‐AS1). (H) Masson staining of aortic sinus (ADV‐OE‐CYP1B1‐AS1). (I) Oil Red O staining of aortic sinus (ADV‐OE‐CYP1B1‐AS1). (J) HE staining of aorta (ADV‐OE‐CYP1B1‐AS1). (K) Masson staining of aorta (ADV‐OE‐CYP1B1‐AS1). (L) Oil Red O staining of aorta (ADV‐OE‐CYP1B1‐AS1). (M–R) Quantitative analysis of plaque composition. (M, P) Relative plaque area in aortic sinus (M) and aorta (P) (ADV‐CON vs. ADV‐OE‐CYP1B1‐AS1). (N, Q) Relative plaque fibrosis in aortic sinus (N) and aorta (Q) (ADV‐CON vs. ADV‐OE‐CYP1B1‐AS1). (O, R) Relative plaque lipid content in aortic sinus (O) and aorta (R) (ADV‐CON vs. ADV‐OE‐CYP1B1‐AS1). (S–V) Transmission electron microscopy (TEM) of aortic sinus. (S, T) Representative TEM images (ADV‐CON). (U, V) Representative TEM images (ADV‐OE‐CYP1B1‐AS1) (p < 0.05). [file JCMM-30-e71066-s010.jpg]
